# Beyond detoxification: Pleiotropic functions of multiple glutathione S-transferase isoforms protect mice against a toxic electrophile

**DOI:** 10.1371/journal.pone.0225449

**Published:** 2019-11-20

**Authors:** Kelsey A. Behrens, Leigh A. Jania, John N. Snouwaert, MyTrang Nguyen, Sheryl S. Moy, Andrey P. Tikunov, Jeffrey M. Macdonald, Beverly H. Koller

**Affiliations:** 1 Curriculum in Toxicology & Environmental Medicine, University of North Carolina at Chapel Hill, Chapel Hill, North Carolina, United States of America; 2 Department of Genetics, University of North Carolina at Chapel Hill, Chapel Hill, North Carolina, United States of America; 3 Carolina Institute for Developmental Disabilities and Department of Psychiatry, University of North Carolina at Chapel Hill, Chapel Hill, North Carolina, United States of America; 4 Department of Biomedical Engineering, University of North Carolina at Chapel Hill, Chapel Hill, North Carolina, United States of America; National Institutes of Health, UNITED STATES

## Abstract

Environmental and endogenous electrophiles cause tissue damage through their high reactivity with endogenous nucleophiles such as DNA, proteins, and lipids. Protection against damage is mediated by glutathione (GSH) conjugation, which can occur spontaneously or be facilitated by the glutathione S-transferase (GST) enzymes. To determine the role of GST enzymes in protection against electrophiles as well as the role of specific GST families in mediating this protection, we exposed mutant mouse lines lacking the GSTP, GSTM, and/or GSTT enzyme families to the model electrophile acrylamide, a ubiquitous dietary contaminant known to cause adverse effects in humans. An analysis of urinary metabolites after acute acrylamide exposure identified the GSTM family as the primary mediator of GSH conjugation to acrylamide. However, surprisingly, mice lacking only this enzyme family did not show increased toxicity after an acute acrylamide exposure. Therefore, GSH conjugation is not the sole mechanism by which GSTs protect against the toxicity of this substrate. Given the prevalence of null GST polymorphisms in the human population (approximately 50% for GSTM1 and 20–50% for GSTT1), a substantial portion of the population may also have impaired acrylamide metabolism. However, our study also defines a role for GSTP and/or GSTT in protection against acrylamide mediated toxicity. Thus, while the canonical detoxification function of GSTs may be impaired in GSTM null individuals, disease risk secondary to acrylamide exposure may be mitigated through non-canonical pathways involving members of the GSTP and/or GSTT families.

## Introduction

The integrity of an organism, spanning from the simplest single-cell life forms to mammals, depends on its ability to protect its structural subunits from damage caused by both strong and weak electrophiles; these electrophiles include those generated through metabolic processes as well as those that are ubiquitous in the environment. Most life forms have addressed this challenge with a multifaceted response, including the production of high levels of intra- and extracellular glutathione (GSH), a potent nucleophile that spontaneously conjugates to a broad range of electrophiles. In addition, virtually all aerobic bacteria and eukaryotes have evolved glutathione *S-*transferase (GST) enzymes, which are capable of facilitating GSH conjugation to these electrophiles. The GST genes have been identified in most species, and some have been shown to take on specialized functions within the species. The importance of these enzymes in the protection of organisms from environmental challenges is perhaps best underscored by the rapid evolutionary changes in GST expression and structure in response to new environmental stresses. For instance, GSTs have been implicated in insect resistance to 1,1,1- trichloro-2,2-bis-(*p*-chlorophenyl)ethane (DDT), and they can also be induced in plants by pathogens, drought conditions, or herbicide exposure [1]. Interestingly, the human population carries genetic polymorphisms in several highly expressed GSTs, and numerous studies have associated the variations in these genes with increased disease risk [2–5]. However, a mechanistic understanding of both the evolutionary driving force that has resulted in the high frequency of GST polymorphisms in most human populations and the consequence of these null alleles in humans is lacking and is hindered in part by our limited understanding of the various functions/activities of these enzymes *in vivo*.

The human and mouse cytosolic GST superfamilies contain 17 and 22 genes, respectively, which have been divided into the following 7 classes: alpha, mu, pi, sigma, theta, zeta, and omega [2, 6]. GSTs within a class share at least 40% of their sequence identity, but there is less than a 30% similarity between the amino acid sequences of different families [7]. Despite this between-class variation, all GSTs follow the same structural pattern. All GSTs are dimeric, and each subunit contains 2 domains. Domain 1, located in the N-terminal region, is highly conserved across all GSTs, and it provides most of the amino acid residues for GSH binding. In contrast, domain 2 is located in the C-terminal region; this region is more varied across families, and differences in the C-terminal region are believed to be responsible for the different substrate specificities between GST families. For instance, the GSTM family has a “Mu loop” located in this domain that results in a deeper active site, as compared to the other GST families [1].

Mice lacking individual or multiple members of these gene families have been used to address the role of GSTs in the pathophysiology of disease and in protection from damage secondary to xenobiotic exposure [6, 8–10]. However, because of potential overlap in the function of the various GST enzymes and a poor understanding of how the functions that have been assigned to these enzymes *in vitro* extrapolate to *in vivo* exposures, progress has been slow. Further complicating our ability to understand these enzymes is the fact that, in addition to facilitiating metabolism through GSH conjugation, GSTs have also been shown to have additional functions, such as participating in non-enzymatic “ligandin” binding or in protein S-glutathionylation [10–15].

Here, we examine the impact of loss of multiple and individual Gst genes on the metabolism and toxicity of the enviromental electrophile acrylamide and its carcinogenic metabolite glycidamide. Specifically, we used previously described mouse lines lacking one, two, or all three of the GSTP, GSTM, or GSTT gene families because these families harbor common polymorphisms in the human population and have been studied for their potential relation to disease risk or altered response to pharmaceutical treatment [2, 5, 16]. The highest human exposures to acrylamide are occupationally-related and result in neuropathic symptoms such as numbness in the hands and feet, muscular weakness, and peeling skin [17]. However, the ubiquitous presence of acrylamide in heated carbohydrate-rich foods such as bread, potato chips, and French fries [18, 19] has raised public concern; although a link between dietary acrylamide consumption and cancer has not been definitively made, significant research efforts are nonetheless being made to develop new technologies to mitigate acrylamide levels in food [20, 21]. Dietary acrylamide exposure is not limited to humans; autoclaving a standard rodent diet has been shown to increase the acrylamide levels 14-fold, thus leading to a substantial acrylamide exposure in research laboratories using this heat sterilization technique [22]. While the metabolism of acrylamide in mammals has been extensively studied, the contribution of the GSTs to this process and the possible mechanisms by which these enzymes can protect against daily exposure to consumption of this toxicant is not known [23–25]. Thus, the possible impact of variation in the repertoire of GSTs that are present in the population cannot be assessed.

## Results

### GST expression and activity in the liver of mice lacking the GSTP, GSTM, and GSTT families

**Fig 1:**
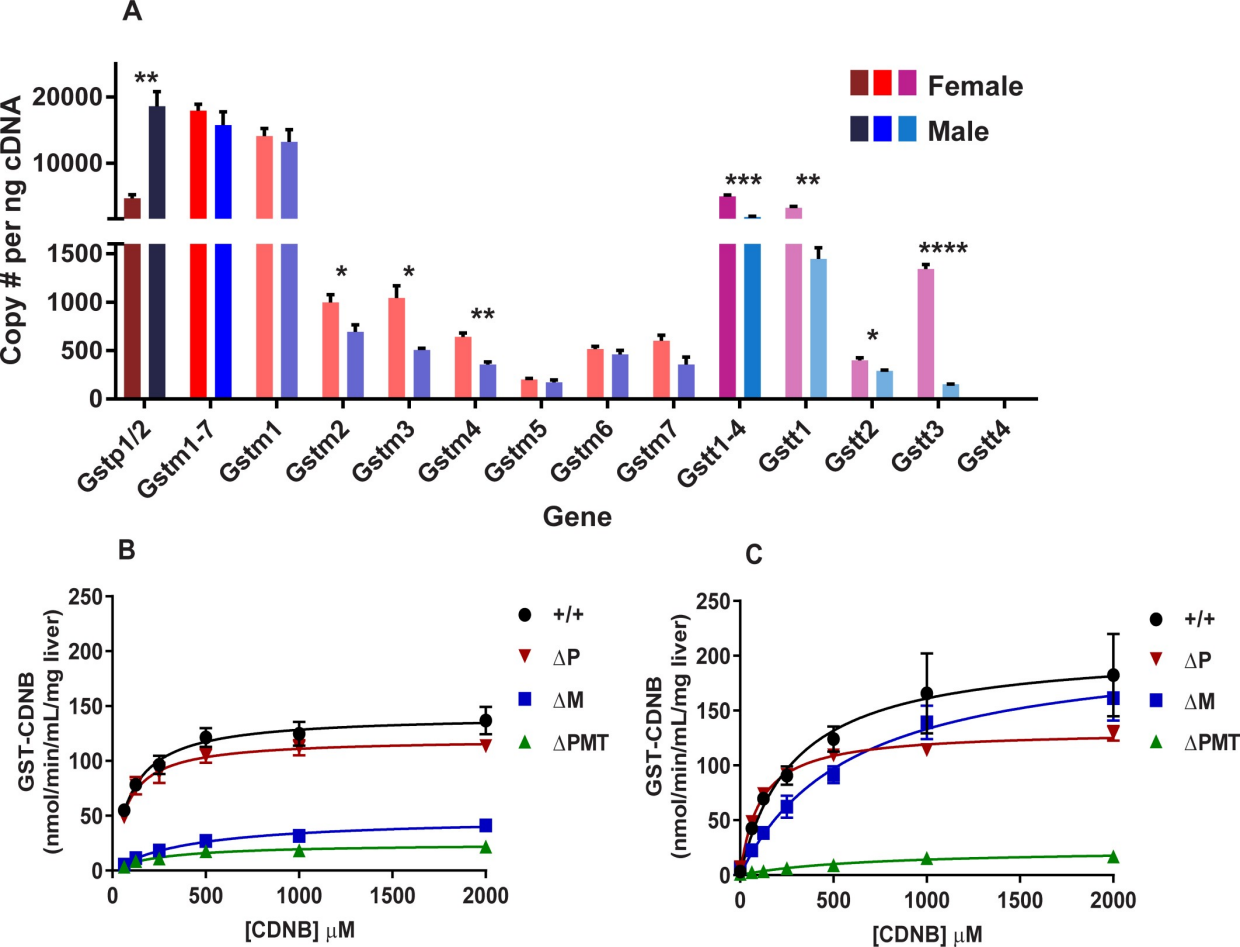
Hepatic GST expression and activity differ between male and female mice. Digital droplet PCR quantification of mRNA copy number of the genes from the GSTM, GSTP, and GSTT families (A) in livers of female and male mice. Numbers of GSTP1/2 transcripts are shown in dark red (female) or dark blue (male). For the GSTM (*Gstm1-7*) family, we first show copies of transcripts for the entire family (red: female; blue: male), followed by copy number for each individual gene within the family (light red: female; light blue: male). For the GSTT (*Gstt1-4*) family, we first show total copy number for the entire family (magenta: female; teal: male), and the copy numbers of the individual genes within this family are represented by a lighter variation of these colors. GST activity towards the substrate CDNB in liver homogenates from female (B) and male (C) livers with different Gst genotypes. Data represent means ± S.E.M.; n = 3. Data analyzed by unpaired t-test between sex for each gene; * p < 0.05; ** p < 0.01; *** p < 0.001; **** p < 0.0001.

As the contribution of a given GST enzyme to xenobiotic metabolism is determined not only by its activity towards a substrate but also by its level of expression, we first evaluated gene expression of the various members of the GSTM, GSTP, and GSTT families. RNA was prepared from the livers of adult male and female 129S6 mice, and copies of mRNA for each gene were determined by digital PCR. Consistent with previous reports, sexual dimorphism in the expression of these genes was very apparent [26]. Approximately 36,000 GST transcripts were observed in the cDNA from the male liver, while only 28,000 copies were observed in samples from female mice. Overall, the GSTM gene family was expressed at a similar level in males and females, with *Gstm1* being the primary transcript expressed at equal levels in both sexes. Although the expression levels of *Gstm2-7* were much smaller, females did have higher transcript levels of *Gstm2*, *Gstm3*, and *Gstm4* relative to males. The difference between males and females in total GST transcripts largely reflected an increase in *Gstp1/2* expression, with only 5,000 copies present in females, as compared to 18,000 in male mice. In contrast, the total number of transcripts in the GSTT family was higher in females than in males, which was primarily attributable to the increased number of *Gstt1* transcripts in the livers of females (3,000 copies) as compared to males (1,000 copies), although females also had increased levels of *Gstt2* and *Gstt3* transcripts (**Fig 1A**).

We next asked whether the single family GSTM and GSTP knock out mouse lines (ΔM and ΔP, respectively) can be used to assign GST activity to a specific gene family. In comparing the hepatic expression of the remaining GST enzymes in the single family knockouts, we observed little to no compensatory upregulation in the knockouts compared to wild-type (WT) mice (**S1 Fig**). Although we observed increases in *Gstm3*, *Gstm4*, *and Gstt2* expression in the ΔP mice compared to WT, the increases themselves were modest (not exceeding 1.6-fold) and comprise a very small percentage (<2% each) of the total GST gene pool. To measure enzymatic GST activity, we used the test compound 1-chloro-2,4 dinitrobenzene (CDNB), as most of the GST families are believed to enzymatically conjugate GSH to this compound [27]. Similar to previous reports, CDNB metabolism by the liver S9 fractions obeyed Michaelis-Menten kinetics (**Fig 1B and 1C**) [28]. Perhaps not surprisingly, given the higher expression of GST transcripts in the male liver, S9 fractions prepared from male mice displayed a higher V_max_ than those prepared from females (**S1 Table**). However, in addition to the higher V_max_, the K_m_ towards CDNB in the S9 fractions from male mice was almost three times higher than that of female mice. Deletion of the GSTT locus had no measurable impact on the kinetics of CDNB metabolism (**S2 Fig**). This is not surprising, since CDNB is known to be a poor substrate for GSTT [29]. In female mice, the loss of either the GSTP1/2 or the GSTM families resulted in a decreased V_max_, but the difference relative to wild-type was more substantial in the ΔM mice than in ΔP mice. In addition to the dramatic reduction in V_max_, loss of the GSTM gene family resulted in an increased K_m_ towards CDNB, indicating that in females the GSTM enzymes are primarily responsible for the activity towards this substrate. A different pattern of changes in metabolism was observed in mutant male mice. Consistent with the higher expression of *Gstp1/2* in this sex, V_max_ was significantly reduced in mice lacking these genes. However, loss of GSTM genes resulted in no statistically significant changes in these enzymatic parameters, although modest increases were noted for both V_max_ and K_m_ in the ΔM males. The fact that differences in the kinetics of metabolism become apparent on evaluation of animals lacking the individual gene families underscores their potential usefulness in assignment of less well studied GST substrates.

### GSTs protect against genotoxicity of both acrylamide and glycidamide

**Fig 2:**
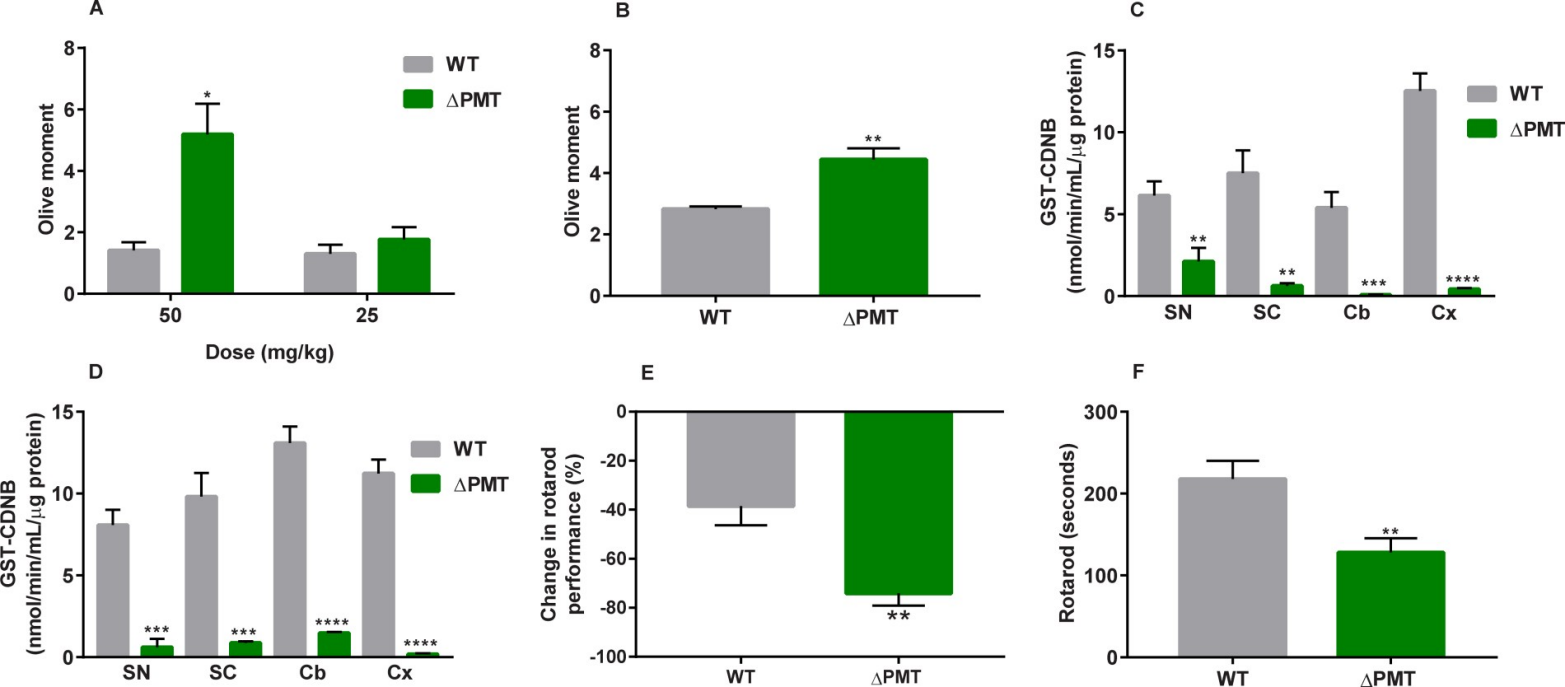
ΔPMT mice are susceptible to the genotoxic and neurotoxic effects of acrylamide and glycidamide. (A, B) DNA damage in liver measured by Comet assay 24 hours after a single injection of (A) 50 or 25 mg/kg acrylamide or (B) 50 mg/kg glycidamide to male mice. DNA damage is measured as Olive Moment. Each mouse was analyzed in duplicate slides, where each slide contained >50 comets. (C, D) GST enzyme activity in nervous tissue of wild-type and ΔPMT female (C) and male (D) mice. (E) Percent change in rotarod performance relative to baseline in male mice after treatment with 200 ppm acrylamide in drinking water for 21 days. (F) Decreased rotarod performance of ΔPMT male mice compared to wild-type after treatment with 50 ppm acrylamide in drinking water for 5.5 weeks. ACR = acrylamide, SN = sciatic nerve, SC = spinal cord, Cb = cerebellum, Cx = cortex. Data analyzed by unpaired t-test between genotypes. Data represent means ± SEM, n = 4–6 (A, B), n = 5 (C, D), n = 10 (E), and n = 6–9 (F); * p < 0.05, ** p < 0.01; *** p < 0.001; **** p < 0.0001.

In contrast to CDNB, very little is known regarding the contribution of GSTs to metabolism and protection of the organism from environmental electrophiles such as acrylamide. To establish a role for GSTs in metabolism of acrylamide, we focused on DNA damage as a biomarker of acute exposure. DNA damage by acrylamide is thought to occur primarily through its phase I metabolism to the epoxide glycidamide, which then forms adducts with DNA [30, 31]. We therefore evaluated the contribution of enzymatic GST metabolism in protection against DNA damage after acute acrylamide and glycidamide exposure. Twenty-four hours after a single i.p. injection of acrylamide or glycidamide, liver samples were evaluated by the Comet assay. A single administration of acrylamide resulted in increased genotoxicity in the ΔPMT mice compared to WT mice, but this effect was dose-specific and only occurred after a 50 mg/kg acrylamide exposure and not at a 25 mg/kg dose (**Fig 2A**). In addition to displaying decreased protection against acrylamide-induced genotoxicity, ΔPMT mice also showed increased DNA damage after glycidamide (50 mg/kg b.w.) exposure (**Fig 2B**).

### Increased sensitivity of ΔPMT mice to the subchronic neurotoxic effects of acrylamide

Another well-documented outcome of acrylamide exposure is peripheral neuropathy, particularly after chronic and subchronic exposures. Unlike genotoxicity, the neurotoxic effects are thought to be mediated largely by acrylamide, although some contributions of glycidamide to neurotoxic endpoints have been shown [32–34].

To determine whether GSTs have a role in neuroprotection, we first measured GST enzyme activity towards CDNB in the sciatic nerve, spinal cord, cerebellum, and cortex in acrylamide-unexposed WT and ΔPMT mice. In all instances, ΔPMT mice of both sexes showed severely reduced GST activity in these tissues compared to WT mice (**Fig 2C and 2D**). This reduced nervous tissue GST activity, as well as the previously described decrease in hepatic GST activity, indicates that ΔPMT mice may also be more sensitive to the neurotoxic effects of acrylamide. To test this hypothesis, we measured rotarod performance in WT and ΔPMT mice before and after treatment with acrylamide. The rotarod test was used because it is one of the most well-defined methods for measuring acrylamide-induced peripheral neuropathy [35–37]. Mouse rotarod performance did not differ by genotype before acrylamide exposure (WT: 182 ± 23.7 seconds vs. ΔPMT: 166 ± 19.8 seconds, means ± SEM). However, after acrylamide was administered through drinking water (200 ppm) for 3 weeks, the decline in rotarod performance in the WT group (-36%) was less than that of the ΔPMT group (-74%), thus indicating that the effect of acrylamide exposure on peripheral neuropathy was greater in the ΔPMT mice (**Fig 2E**). To assess GST protection against neuropathy at lower levels of acrylamide exposure, mice were exposed to 50 ppm acrylamide in drinking water at a young age (2–3 weeks old) for 5.5 weeks. Even at these lower levels of exposure, rotarod performance in ΔPMT mice was significantly lower than that of WT mice (>40% lower) (**Fig 2F**). Together, these studies show that GSTs protect against the peripheral neuropathy of acrylamide, regardless of the dose that we tested.

### GSTP, GSTM, and GSTT protect against systemic toxicity of acrylamide

**Fig 3:**
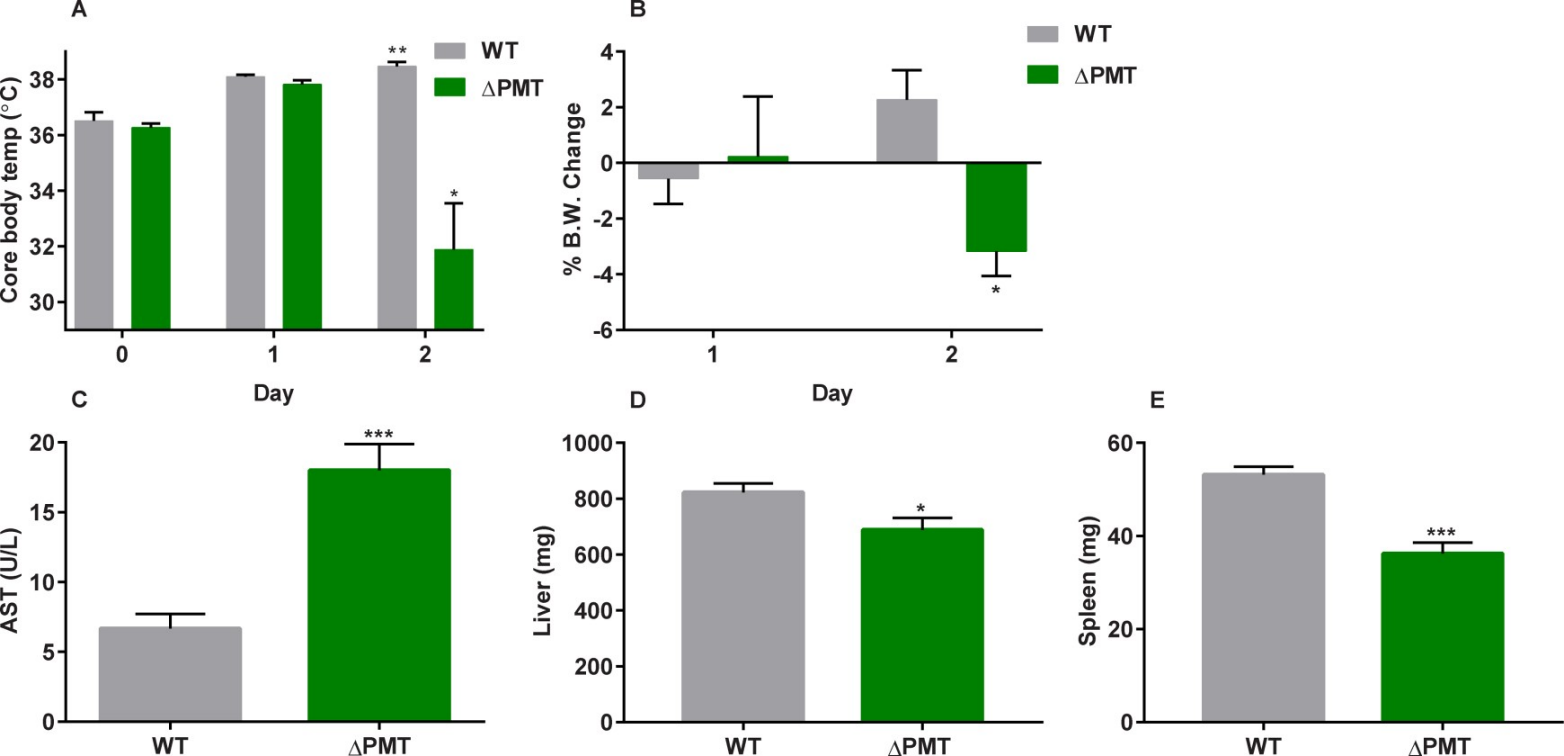
ΔPMT mice display an increased sensitivity to the acute systemic toxicity of acrylamide. Female wild-type and ΔPMT mice received two acrylamide injections (50 mg/kg i.p.) once every 24 hours over a 2-day period. Day 0 represents pre-exposure values. Core body temperature measurements (A) over the exposure period and body weight change (B) were recorded during this period. Following necropsy 24 hours after the second injection, AST (C), liver weights (D), and spleen weights (E) were also recorded. Data represent means ± S.E.M.; n = 6. Data were analyzed by paired t-test (Day 0 vs. Day 2) within each genotype (A, B) or by t-test between genotypes on Day 2 (C–E); * p < 0.05; * p < 0.01; *** p < 0.001.

We next determined if mice lacking individual gene families could be used to identify the GST enzymes mediating this protective action against acrylamide using well-established models of acute toxicity. Our initial goal was to identify not only an appropriate dose regime but also additional physiological and biochemical markers that could be useful in assignment of protection against acrylamide. To accomplish this, we first administered acrylamide at 50 mg/kg b.w. by i.p. injection once every 24 hours, as this dose was previously shown to be tolerated by WT mice for up to 5 days [30, 38]. However, it was apparent after 2 injections that this would not be appropriate for our studies due to the increased sensitivity of the ΔPMT mice to acrylamide, and thus the time course was limited to 2 injections. Twenty-four hours after the second dose, the majority of the ΔPMT mice presented with a decrease in ability to thermoregulate core body temperature and significant weight loss (**Fig 3A and 3B**). The ΔPMT mice were also distinguished by their ruffled fur, trembling, and agitated behavior. Elevated AST levels were measured in the serum collected from the ΔPMT animals (**Fig 3C**). In contrast to AST, no consistent increase in ALT was observed throughout these studies (**S4 Fig**), especially compared to the model hepatotoxicant acetaminophen, suggesting that the AST increase was the result of systemic damage rather than liver specific cytotoxicity [39]. Consistent with this, a survey of expression of numerous genes dysregulated in the liver after oxidative stress and/or exposure to cytotoxic agents found that they were not consistently altered in the ΔPMT mice (**S2 Table**). This and the small magnitude of change in ALT expression made them inappropriate for further assignment of GST function to a single gene family. Despite the lack of a corresponding ALT increase to indicate hepatotoxicity, a modest decrease in liver weight was noted in ΔPMT mice relative to WT (**Fig 3D**). Spleen weights were also measured to determine the potential immunotoxic effects of acrylamide, and decreased spleen weights were measured in ΔPMT mice relative to WT mice (**Fig 3E**). Although raw organ weights are presented in the manuscript, these changes in raw organ weights remained consistent when they were instead calculated as percentage relative to starting body weight (before acrylamide treatment) or ending body weight (at necropsy) (**S5 Fig**). As the observed decrease in spleen weight was not accompanied by a decrease in circulating red blood cell counts, we evaluated the number and composition of white blood cells in the spleen. We chose to examine leukocyte number in the spleen rather than blood leukocytes because of the high variability in blood leukocyte number and composition between individual animals. An evaluation of the total white cell counts and fluorescence-activated cell sorting analysis of splenic leukocytes prepared from acrylamide-treated ΔPMT male mice showed a reduction in the number of T cells, B cells, and macrophages in comparison to both wild-type treated mice and controls. Since acrylamide lowered the numbers of all of the major splenic leukocyte populations, we used spleen weight as a convenient surrogate marker for the acrylamide-mediated decrease in leukocytes (**S6 Fig**). Thus, we concluded that the ΔPMT mice are particularly susceptible to leukopenia after this acute level of acrylamide exposure. The decreased spleen size and leukopenia that we observe are similar to a report of immune suppression in which rats exposed to acrylamide displayed decreased spleen size and cellularity, in addition to decreased circulating blood lymphocytes and impaired humoral and cell-mediated immune responses [40].

### Gastroparesis is a sensitive indicator of acute acrylamide toxicity

**Fig 4:**
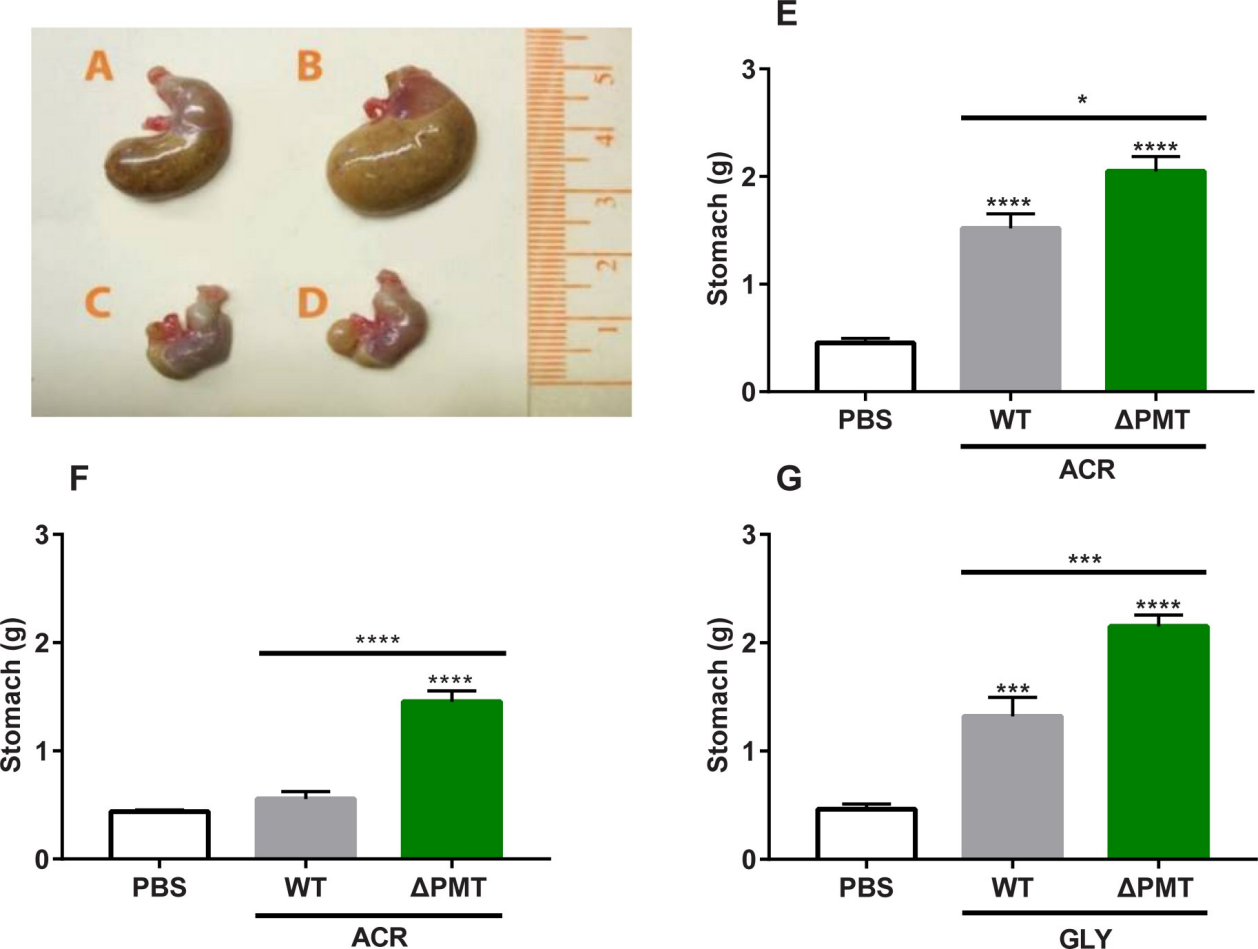
Gastroparesis is a novel, GST-sensitive biomarker of acute acrylamide toxicity. Stomach enlargement of male wild-type (A) and ΔPMT (B) mice 24 hours after a 50 mg/kg dose acrylamide exposure. Stomachs of male wild-type (C) or ΔPMT (D) mice treated with PBS control are provided for reference. Stomach weights were measured after a single 50 mg/kg (E) and 25 mg/kg (F) dose of exposure. (G) Stomach weights of male mice after a single 50 mg/kg exposure to glycidamide. All measurements were taken 24 hours after the injection. ACR = acrylamide, GLY = glycidamide. Data represent means ± SEM; n = 9–18 (E-F) or 6 (G). Data analyzed by one-way ANOVA corrected for multiple comparisons; * p < 0.05; *** p < 0.001; **** p < 0.0001.

Enlarged stomachs were observed in both WT and ΔPMT mice exposed to a single (50 mg/kg) injection of acrylamide (**Fig 4A–4D**). Stomach weights were increased relative to controls in both WT and ΔPMT mice exposed to acrylamide. Importantly, the weight of the ΔPMT stomachs was greater than that of WT mice, indicating that GSTs protect against acrylamide-mediated gastroparesis (**Fig 4E**). This GST protection was more apparent when the dose of acrylamide was reduced to a single exposure of 25 mg/kg, as gastroparesis under this condition was still apparent in the ΔPMT group but was not noted in the WT group (**Fig 4F**). In contrast, increasing the dose of acrylamide to 75 mg/kg obscured the difference between genotypes (**S7 Fig**). Gastroparesis was also observed in both WT and ΔPMT mice after a single 50 mg/kg exposure to glycidamide. Within the glycidamide-exposed group, stomach size in the ΔPMT group was significantly larger than that of WT mice (**Fig 4G**).

Despite the fact that acrylamide exposure resulted in gastroparesis in WT 129S6 mice at exposures commonly used in studies of acrylamide toxicity, we were unable to identify reports of this particular finding by other investigators who exposed mice to similar or higher acrylamide doses at various routes of administration [30, 31, 41]. We hypothesized that our finding may be unique because the strain used for our studies (129S6) is rarely used in toxicological studies. This is supported by our failure to observe gastroparesis in either C57BL/6 or BALB/c mice exposed to 50 mg/kg of acrylamide; only at single exposures of 100 mg/kg did gastroparesis become apparent in the other strains (**S7 Fig**). As all the mice used in our study are co-isogenic 129S6, gastroparesis provides a GST sensitive biomarker for determination of acrylamide mediated tissue damage.

### Protection against high-dose acrylamide exposure cannot be attributed to GSTM in female mice

**Fig 5:**
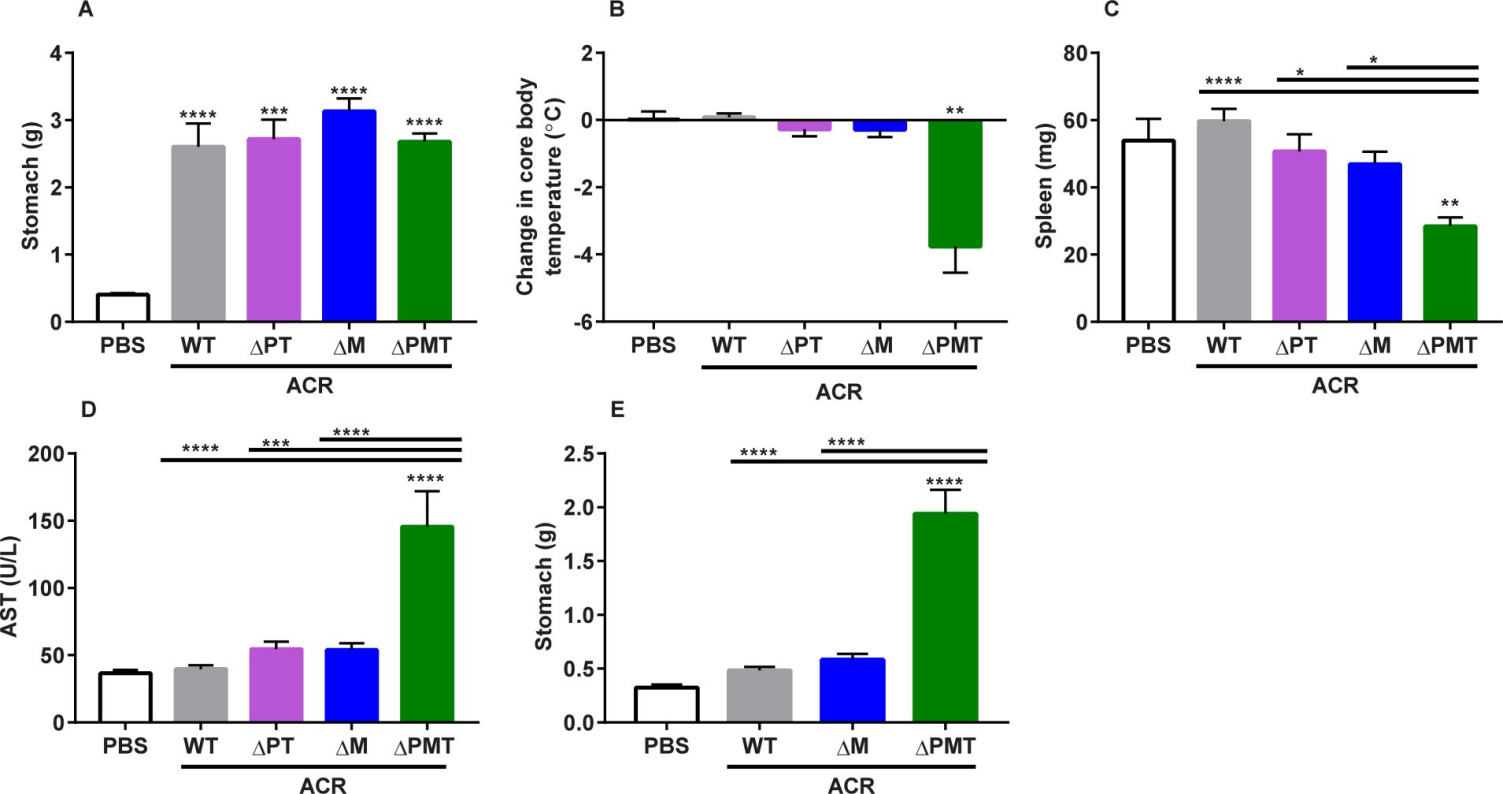
The sensitivity of female ΔPMT mice to acrylamide cannot be assigned to a gene family. (A) Stomach weight, (B) change in core body temperature, (C) spleen weight, and (D) AST levels in female mice exposed to 2 50 mg/kg injections of acrylamide. (E) Stomach weight of female mice exposed to 2 25 mg/kg injections of acrylamide. ACR = acrylamide. Data represent means ± S.E.M.; n = 4–10 (A-D) and n = 3–11 (E). Data analyzed by ANOVA (A, C-E) or paired t-test, relative to starting temperature (B); * p < 0.05; ** p < 0.01; *** p < 0.001; **** p < 0.0001.

The structural differences between GST families support the possibility that a specific GST family, or even a specific enzyme, will preferentially metabolize any given xenobiotic [1]. To determine if protection from acute acrylamide toxicity can be assigned to a specific GST family, we evaluated metabolism in mice lacking only one or two of the GST families. As female mice have low levels of GSTP, a dominant role for the GSTM family in protection from acrylamide should be more apparent in this sex. Therefore, we conducted an experiment in female ΔM mice, which carry a 150 kb deletion in which the *Gstm1-7* genes have been removed. Mice lacking all GSTM genes (ΔM) and mice lacking the GSTP and GSTT families (ΔPT) were exposed for two consecutive days to 50 mg/kg acrylamide, with WT and ΔPMT mice included for comparison. At this exposure, WT mice did not differ from untreated animals with the exception of notable gastroparesis similar in magnitude to the mutant cohorts (**Fig 5A**). The ΔPMT mice showed a dramatic loss of thermoregulation that was not apparent in the ΔM or the ΔPT mice (**Fig 5B**). Both the ΔM and ΔPT mice were protected against leukopenia (low splenic leukocyte numbers) and generalized tissue damage (AST) (**Fig 5C and 5D**).

To utilize gastroparesis as a biomarker in assessing the contribution of the GSTM gene family in acrylamide protection, a second experiment was performed at a lower dose (2 i.p. injections of 25 mg/kg b.w.). At this dose, the only difference that was observed between the GST deficient and WT animals in any of the previously-described biomarkers was gastroparesis, which was only observed in the ΔPMT mice. Similar to WT mice, the ΔM females were largely protected from this acrylamide mediated damage to gastric emptying (**Fig 5E**). The protection of the ΔM mice against acrylamide-mediated gastroparesis shows that protection against acrylamide toxicity cannot be assigned to the GSTM family alone, despite the fact that this is the predominant GST family in the female mouse liver. However, it would be interesting to extend these studies in the future over additional exposure regimes using both the ΔPT and ΔM mice to further investigate the role of the GSTM family over a broad dose range.

### Protection against high-dose acrylamide exposure cannot be attributed to GSTP in male mice

**Fig 6:**
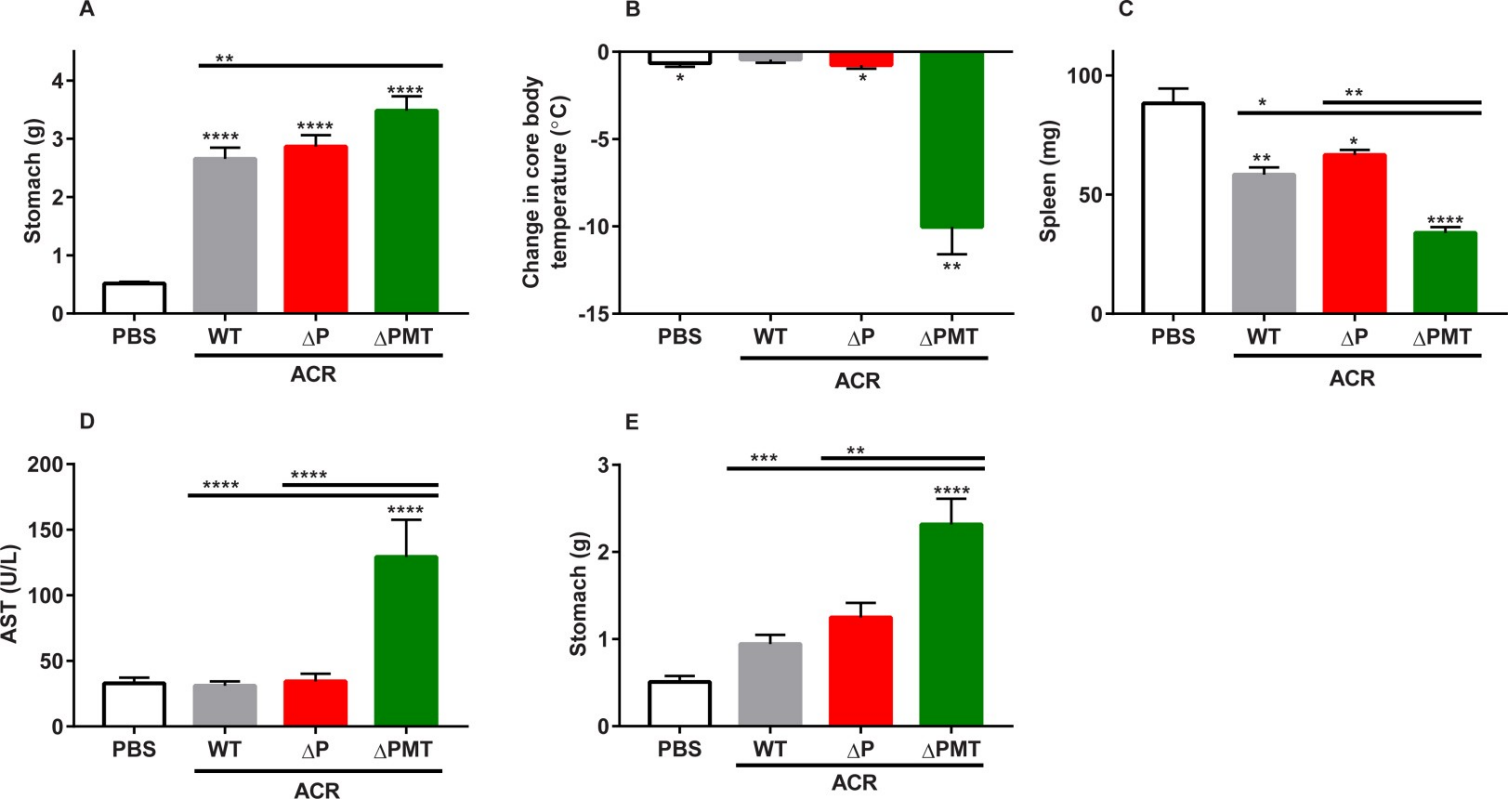
The increased sensitivity of male ΔPMT mice cannot be assigned to GSTP. (A) Stomach weight, (B) change in core body temperature, (C) spleen weight, and (D) AST levels in male mice exposed to 2 50 mg/kg injections of acrylamide. (E) Stomach weight of male mice exposed to 2 25 mg/kg injections of acrylamide. ACR = acrylamide. Data represent means ± S.E.M.; n = 6–11 (A-D) and n = 5–11 (E). Data analyzed by ANOVA (B-E) or paired t-test, relative to starting temperature (B); * p < 0.05; ** p < 0.01; *** p < 0.001; **** p < 0.0001.

We next examined the contribution of GSTP against acute acrylamide toxicity in male mice, due to its high protein expression in the male mouse liver. Male WT, ΔP, and ΔPMT mice were exposed to two consecutive injections of 50 mg/kg bw/day of acrylamide. We again observed that stomach size had increased in all genotypes after acrylamide exposure (**Fig 6A**). Similarly to females, we observed a dramatic drop in core body temperature in ΔPMT male mice that was not observed in the similarly treated WT or ΔP mice (**Fig 6B**). A small decrease in spleen weight was observed in all mice of all genotypes after acrylamide exposure, but the ΔP mice did not show any increased sensitivity over WT in this response. In contrast, the ΔPMT mice had reduced spleen weights compared to the acrylamide-exposed WT and ΔP groups (**Fig 6C**). AST was increased only in the ΔPMT genotype after acrylamide exposure (**Fig 6D**). To examine a possible role for GSTPs in protection against gastroparesis, an additional experiment was performed at a lower acrylamide exposure of 25 mg/kg bw/day. In this experiment, the ΔP mice were significantly protected from acrylamide-mediated gastroparesis (**Fig 6E**). These results demonstrate that, despite the clear adverse effects of this high-dose acrylamide exposure in the ΔPMT group, the sensitivity of ΔPMT mice cannot be attributed solely to loss of GSTP. Therefore, protection from the toxic effects of acrylamide at these levels of exposure cannot be attributed solely to GSTM or GSTP.

### ^1^H NMR evaluation of mercapturic acid metabolites after acrylamide exposure identifies contribution of GSTs to metabolism

**Fig 7:**
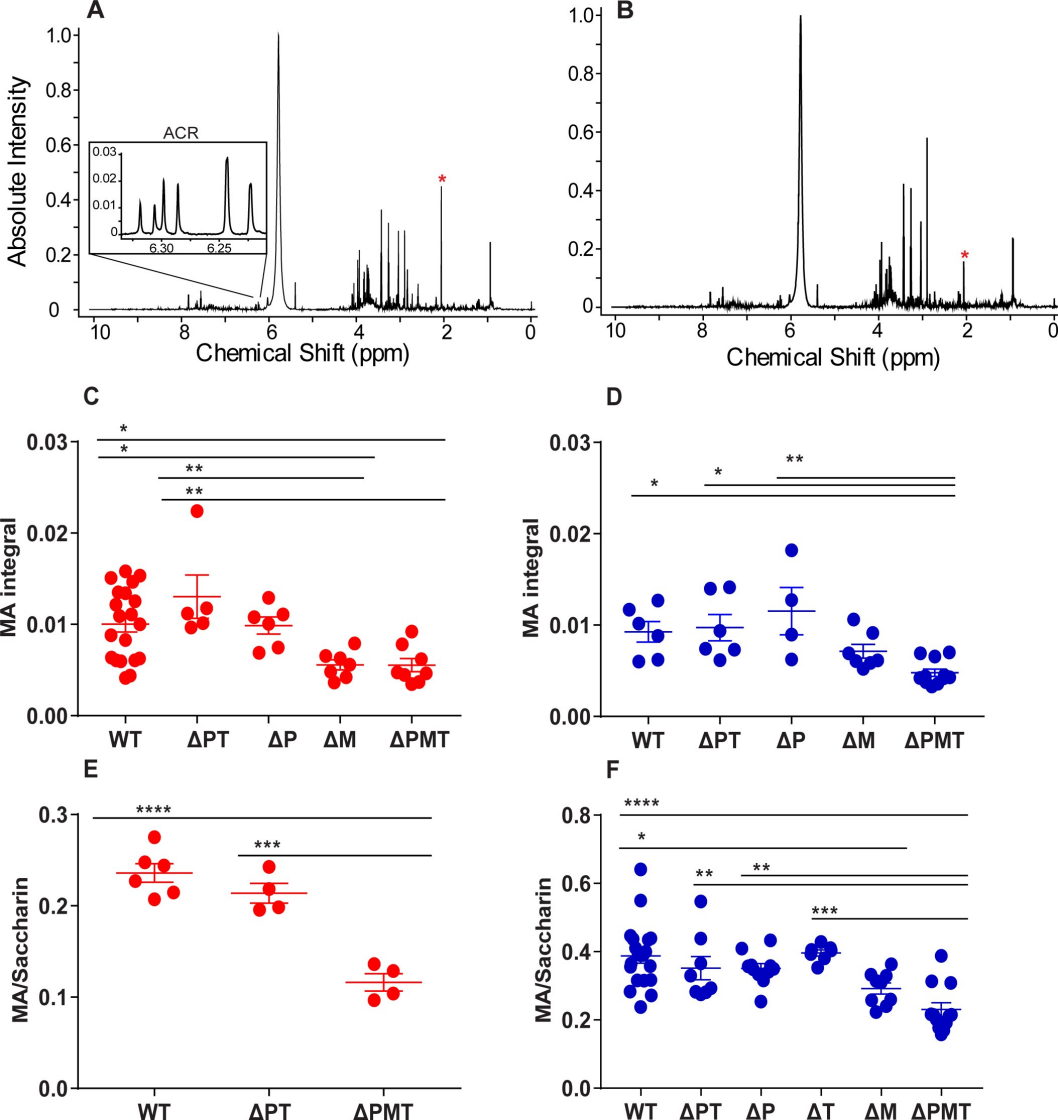
GSTM is primarily responsible for the metabolism of acrylamide to its mercapturic acid. Representative ^1^H NMR spectra of urine collected from male wild type (A) and ΔPMT (B) mice 90 minutes after a single i.p. injection of 50 mg/kg b.w. acrylamide. The acrylamide multiplet is shown in (A), and the MA peaks are indicated by the asterisks (*) in each panel. ^1^H NMR analysis of MA metabolites (C, D) of urine 90 minutes after exposure to 50 mg/kg acrylamide in female (C) and male (D) mice. Saccharin-normalized MA metabolites in urine of additional cohorts of female (E) and male (F) mice 90 minutes after a coinjection of 50 mg/kg ACR and saccharin. Data represent means ± S.E.M., n = 4–20 (C, D); n = 4–20 (E, F); * p < 0.05, ** p < 0.01, *** p < 0.001 **** p < 0.0001, analyzed with one-way ANOVA corrected for multiple comparisons.

Enzymatic GSH conjugation by GSTs to electrophiles is the first step in the generation of mercapturic acids (MAs) for renal excretion. We therefore asked whether the enzymatic conjugation of acrylamide and its metabolite glycidamide could be assigned to one GST gene family. To accomplish this, we utilized ^1^H NMR, a method that has been previously used to detect the urinary MA metabolites of acrylamide and glycidamide [42]. The use of ^1^H NMR to quantitate urinary MA levels after acute acrylamide exposure would allow us to determine the contribution of GSTs to this electrophile-glutathione conjugation *in vivo*. WT and ΔPMT male mice received a single bolus injection of 50 mg/kg, and urine was collected over the next 90 min. Acrylamide-exposed mice displayed a peak at 2.06 ppm that is representative of the MA metabolites that are derived from both acrylamide and glycidamide. Relative to WT mice, the MA peak was notably smaller in the ΔPMT group, supporting a role for the GSTM/P/T enzymes in GSH conjugation (**Fig 7A and 7B**). The measurable MA peak in the ΔPMT mice likely represented non-enzymatic GSH conjugation.

### Contribution of GSTP and GSTM gene families to GSH conjugation of acrylamide

Female WT, ΔPT, ΔP, ΔM, and ΔPMT mice received a bolus dose of 50 mg/kg acrylamide, and urine was collected over the next 90 min. As expected, MA levels were significantly reduced in the ΔPMT mice compared to WT animals. The presence of GSTM and GSTT (ΔP) or GSTM alone (ΔPT) was sufficient to increase the urinary MAs to their WT levels. In addition, the ΔM females showed MA levels that were significantly reduced compared to WT and were more similar to those observed in the ΔPMT animals (**Fig 7C**), suggesting that in female mice, MA formation after exposure to high doses of acrylamide is almost entirely mediated by the GSTM gene family.

To determine the contribution of GSTP to MA formation, we performed a similar experiment with male mice. When male mice were subjected to the same acrylamide dosing scheme, we observed a similar decrease in MA excretion of ΔPMT mice relative to WT. MA formation in both the ΔP and ΔPT groups was significantly higher than that observed in the ΔPMT mice, indicating that the loss of either of these two gene families did not result in the deficiency in metabolism that was observed in the ΔPMT mice. Although MA levels in the ΔM mice were slightly higher than those of the ΔPMT group, this difference was not significant. Therefore, while the expression of only the GSTM family (in ΔPT mice) or the expression of both the GSTM and GSTT families (in ΔP mice) resulted in a significantly increased capacity for acrylamide metabolism over that of the ΔPMT mice, the expression of both the GSTP and GSTT families (in ΔM mice) did not (**Fig 7D**). Thus, in male mice, GSTM was also the only one of the three gene families to which we could assign a role in MA formation.

To verify our findings, we conducted additional experiments with cohorts of female and male WT and mutant mice in which we normalized the urinary MA levels to those of a coinjected non-metabolizable compound. We reasoned that normalization to such a compound would address the sample-to-sample variation that may be caused by the dilution of acrylamide/MA in urine that could result if the mouse voided its urine immediately prior to the experiment. In addition, this normalization could correct for any difference in urine formation during the 90 min experimental interval. Sodium saccharin (sacc, 50 mg/kg body weight) was chosen as a standard because a number of studies indicate that it is not metabolized [43, 44]. Moreover, we found it to be rapidly excreted in the urine, and its location at 7.81–7.92 ppm does not interfere with any acrylamide-derived peaks (**S8 Fig**). As expected, in females the MA/sacc ratio in the ΔPMT group was reduced relative to WT, whereas the ΔPT showed a significantly higher MA/sacc ratio relative to the ΔPMT group (**Fig 7E**). Our study of male mice was extended to include mice of all genotypes. Again, the MA/sacc ratio was reduced in the ΔPMT group relative to WT. In contrast, the MA/sacc ratios for the ΔP, ΔT, and ΔPT groups were significantly higher than those of the ΔPMT group, indicating that the GSTM family is the only one of these three enzyme families that is needed for enzymatic MA formation. Although a small increase was observed in the MA/sacc ratio of the ΔM mice relative to the ΔPMT group, it did not achieve significance. Moreover, the MA/sacc ratio of the ΔM group was significantly reduced compared to that of WT mice. Both of these findings indicate that GSTM is the primary gene family to contribute to MA formation in male mice (**Fig 7F**). Thus, the ΔM mice are protected from acute tissue damage secondary to acrylamide exposure despite the dominant role of GSTM in formation and excretion of the MA of this toxicant.

## Discussion

Any electrophile that is subject to GSH conjugation by GSTs can also undergo spontaneous conjugation with this nucleophile under some conditions [45].GSH conjugation is the first step in the excretion of electrophiles, after which the GSH-electrophile conjugate is processed further into a MA derivative, which is then excreted. The urinary acrylamide- and glycidamide-derived MA metabolites have previously been shown to correlate well with the area under the time concentration curves for both acrylamide and glycidamide, which means that urinary MA measurement can be used as a parameter to estimate internal acrylamide or glycidamide exposures [46]. However, the measurement of urinary MA metabolites does not allow us to determine whether the initial GSH conjugation step occurred enzymatically or spontaneously. Thus, although it is clear that cells are protected against acrylamide by GSH conjugation, the contribution of GST enzymes to this process has not been established. Previous work showed that GSTs could mediate acrylamide-GSH conjugation in both brain and liver lysates [47, 48]; however, the *in vivo* evidence has been sparse. Our study shows that GSTs do contribute *in vivo* to acrylamide conjugation, as MA formation was largely compromised in the mice lacking the GSTP/M/T gene families. However, we did still observe a residual MA peak in these GST-compromised mice, which is indicative of spontaneous conjugation to glutathione or to metabolism by a GST belonging to a different family, such as GSTA. What is not clear from our studies is how the dose of acrylamide and the expression of GSTs in a cell alter the relative contribution of enzymatic versus nonenzymatic formation of MAs.

To examine the enzymatic metabolism of acrylamide at acute exposures, we used ^1^H NMR to quantify the levels of urinary MAs in mice of different genotypes. This method has previously been used to analyze urine after chronic exposure in rats, although in that study it was part of comprehensive metabolomics analysis and not specifically applied to quantification of acrylamide metabolites [42]. Other studies have quantified urinary acrylamide-derived metabolites in rodents and humans using ^13^C NMR and/or LC-MS/MS period following an acute administration of acrylamide [23–25, 49]. We reasoned that ^1^H NMR could provide a means for measuring formation of MA immediately after exposure to acute acrylamide. If this was the case, MA formation could be assigned to a specific gene family. Our study supports a preferential role for the GSTM family in the enzymatic detoxification of acrylamide. We failed to assign this function to GSTP, even though this is the predominant liver isoform in males. In addition, we demonstrated the utility of sodium saccharin coadministration as a method for the normalization of urinary metabolites. To our knowledge, this is the first time that saccharin has been used as a normalization parameter that can control for differences in urinary volume and timing of bladder emptying. We demonstrated the viability of this method and anticipate it to be useful in future experiments of the metabolism of other compounds beyond acrylamide.

Certain electrophiles have been identified as being metabolized by multiple GST families. In fact, the substrate promiscuity of CNDB has made it a tool for the study of the combined activity of the GSTP and GSTM enzymes in tissues [2]. Using liver homogenates, we showed a similar overlap in CDNB metabolism by GSTP and GSTM; however, we also demonstrated a differential sensitivity of these two families for CDNB based on the K_m_. In contrast to CDNB, other compounds have been shown to be preferentially metabolized by a particular GST family. For example, atrazine is widely believed to be metabolized almost exclusively by GSTP, whereas the metabolism of trans-stilbene oxide has been attributed specifically to GSTM1 [50, 51]. Clearly, metabolism of a compound by a single gene family has implications for exposure risk, particularly in the case of the GSTM and GSTT families, where a high percentage of the population carries null alleles for the primary enzyme of each family.

While our demonstration of perhaps an exclusive role for the GSTM family in the detoxification of acrylamide could have important implications for individuals who are homozygous for GSTM1*0, the concern raised by this finding is tempered by our failure to observe any increased sensitivity in the ΔM mice *in vivo* at high levels of exposure. This suggests that a diminished capacity for GSTs to conjugate glutathione may not result in a decreased protection against the harmful effects of this electrophile. Rather, the different GST families appear to be redundant in their protection against acrylamide toxicity, but this redundancy could also stem from the different mechanisms of protection, i.e., through detoxification (GSH conjugation) or alternative non-detoxification functions.

There are several possible explanations for the disparity between the severely impaired acrylamide metabolism and *in vivo* acrylamide toxicity in the ΔM mice. The ΔM mice, which excrete levels of MAs that are similarly decreased as the ΔPMT group, still express the other GST enzymes, and GSTP in particular has received significant attention for its mechanisms of protection outside of its enzymatic properties [9, 13, 52]. For example, it is well-known that the overexpression of GSTP in certain solid tumors in cancer patients is associated with chemotherapy drug resistance, despite the fact that these chemotherapy drugs are not conjugated by GSTP [12]. One mechanism that has been proposed to explain this protection is the “ligandin” function of GSTP. In this model, GSTs bind to molecules and sequester them but do not catalyze conjugation to GSH. The ligandin binding site (“L-site”) has been identified in human GSTP [11]. Thus, it is possible that GSTP, despite being unable to metabolize acrylamide, sequesters the electrophile, limiting its ability to form both protein and DNA adducts. Although acrylamide is much smaller in size than the ligandin substrates studied to date, we cannot rule out the possibility that GSTs sequester acrylamide through such a mechanism and that this function is not specific to a single family.

Recently there has been a great deal of interest in the S-glutathionylation properties of GSTs, with most studies to date focused on GSTP. S-glutathionylation is a reversible process in which GSH is added to low pK cysteine sulfhydryl or sulfenic acid moieties on proteins as part of cellular signaling or during oxidative/nitrosative stress. This GSH addition can therefore act as a temporary measure to protect these vulnerable sites from damage by reactive oxygen/nitrogen species. GSTP has been shown to mediate the forward process of S-glutathionylation [10, 12, 14, 15, 53]. In addition, acrylamide toxicity has been linked to its capacity to adduct to cysteine sulfhydryl groups on proteins [41, 54]. Therefore, S-glutathionylation by GSTP or other GSTs could limit the formation of acrylamide protein adducts, limiting cellular damage. However, the question of whether protein S-glutathionylation is protective at the same proteins and cysteine residues that are targeted by acrylamide remains to be determined.

In summary, our studies suggest that the function of GSTs in protecting cells against both endogenous and exogenous electrophiles is complex and involves other pathways beyond glutathione conjugation that limit toxicity. While a specific enzyme might be assigned to the formation of the MA, other GSTs can provide protection against cytotoxic actions of the electrophile. The mechanisms by which these non-detoxification pathways limit toxicity, as well as their relative contribution to limiting toxicity at various exposure levels, deserves additional investigation. Future studies using the lines described here as well as additional mouse lines in which various GSTs have been engineered to lack a specific function should provide a means of addressing these questions for various exposure models.

## Materials and methods

### Mouse lines and reagents

Mice for these studies were bred and maintained in specific pathogen-free housing in ventilated caging. Generation of the co-isogenic 129S6/SvEvTac GST null line has been previously described [16]. These genetically modified mouse lines include GSTM-deficient (ΔM) mice, which carry a 150 kb deletion of the *Gstm1-7* genes, GSTP-deficient (ΔP) mice, which carry a 40 kb deletion in the *Gstp1-3* genes, and GSTT-deficient (ΔT mice, which carry a 65 kb deletion in the *Gstt1-4* genes. The experiments also included triple knockout mice lacking all three families (ΔPMT) as well as mice lacking the GSTP and GSTT families (ΔPT). To minimize environmental differences contributing to phenotype, mice of various genotypes were generally grouped and co-housed together at weaning. Studies were conducted in accordance with the National Institutes of Health Guide for the Care and Use of Laboratory Animals, and these studies were also approved by the Institutional Animal Care and Use Committee (IACUC) at the University of North Carolina–Chapel Hill. During the experiments, all mice were monitored daily and were removed from the experiment if they approached any of the humane endpoints defined by the IACUC at the University of North Carolina–Chapel Hill [55]. Acrylamide, glycidamide, Dulbecco’s phosphate-buffered saline (DPBS), Hank’s balanced salt solution (HBSS), EDTA, L-glutathione reduced, 1-chloro-2,4,-dinitrobenzene (CDNB), sodium azide, sodium saccharin, and 3-(Trimethylsilyl)propionic-2,2,3,3-d_4_ sodium salt (TSP) were purchased from Sigma Aldrich. Dimethyl sulfoxide (DMSO) was purchased from Corning.

### RNA extraction and gene expression analyses

mRNA was extracted from tissues with RNA-bee (Tel Test, Inc.), and cDNA was generated with the High Capacity cDNA Reverse Transcription kit (Applied Biosystems). For the samples that were analyzed by digital PCR (ddPCR), 10 ng of cDNA were analyzed using ddPCR Supermix (no dUTP, Bio-Rad), commercially available primers/probes, and a Biosystems QX200 Droplet Generator (Bio-Rad). Data were acquired and analyzed using QuantaSoft Software and the QX200 Droplet Reader (Bio-Rad). For the samples that were analyzed by real-time PCR (qPCR), the cDNA was amplified using TaqMan Fast Advanced Master Mix (Applied Biosystems) with commercially available primers (ThermoFisher). Samples were run in duplicate, and the transcript levels for each gene were normalized to those of 18S using the ΔC_T_ method. Fold changes in the acrylamide-treated mice were then normalized to those of the control (PBS-treated) groups using the ΔΔC_T_ method.

### GST-CDNB enzyme activity

Tissues were harvested from mice after transcardial perfusion with DPBS and homogenized in buffer (100 mM KH_2_PO_4_, 2 mM EDTA, pH 7.0). Livers were homogenized at a ratio of 150 mg tissue/1 mL buffer; both sciatic nerves from each mouse were pooled in 0.2 mL buffer; all other tissues (cerebellum, cortex, and spinal cord) were homogenized in 10x buffer/tissue weight. Tissue homogenates were centrifuged at 10,000 g for 15 min at 4°C. Aliquots of homogenates were stored at -80°C until analysis of GST activity was carried out as previously described [56]. Kinetics parameters were calculated using GraphPad Prism version 7.0 software (GraphPad Software, La Jolla, CA).

### Acrylamide/Glycidamide treatment

Mice received 1–2 intraperitoneal (i.p.) injections of the indicated amounts of acrylamide or glycidamide in DPBS or vehicle at a volume of 10 μL/g mouse body weight. Core body temperature was measured using a rectal probe (Physitemp Instruments). For chronic oral exposure, mice first received 50 ppm acrylamide in drinking water *ad libitum* for two days to acclimate mice to acrylamide taste, thus avoiding dehydration secondary to water aversion. Following this acclimation period, mice were exposed to the indicated dose of acrylamide throughout the experimental period.

### Comet assay

Tissues were placed in 4°C buffer (HBSS, Ca^++^ and Mg^++^ free; 20 mM EDTA; 10% DMSO), minced with scissors prior to flash-freezing in liquid nitrogen, and stored at -80°C. For Comet assay analysis, samples were thawed at 23°C, and samples containing ~10,000 cells (5–10 μL cell suspension mixed with 75 μL 0.5% low-melting point agarose) were spread onto microscope slides that had been pre-dipped in 1% agarose. Samples were then lysed and electrophoresed as described [57]. Slides were stained with SYBR Green I Nucleic Acid Gel Stain (ThermoFisher) and viewed at 10x magnification on a light microscope (Olympus BX61). Microscopy images were analyzed, without knowledge of the genotype, using the OpenComet plugin on ImageJ [58].

### Rotarod performance

To measure motor coordination, mice were placed on an accelerating rotarod (Ugo Basile, Stoelting) that was set at an initial speed of 3 rpm and accelerated at a constant rate to 30 rpm at the end of the 300 second trial period. To train/habituate mice to this instrument, animals were subjected to three trial runs. Baseline performance was determined 2 days after training, in which 2 trial periods were given, with at least 40 seconds between each trial. To determine the effects of acrylamide on motor neuron function, an additional rotarod test was performed after the exposure period (either 21 days or 5.5 weeks), followed by a 3-day chemical washout period. Performance was measured as the average time spent on the apparatus, or latency to fall, between the 2 trials. For the 21-day exposure experiment, the post-treatment rotarod performance was calculated as a percentage relative to baseline performance. When no baseline test was performed, rotarod performance was compared directly between experimental groups.

### Plasma separation and clinical chemistry

Blood was collected at necropsy by cardiac puncture and plasma prepared by centrifugation at 3000 rpm for 10 min at 4°C. AST and ALT levels were measured on a Vet Axcel Clinical Chemistry System (Alfa Wassermann).

### Fluorescence-activated cell sorting analysis

Spleens were collected from experimental animals, and splenocytes were stained with markers specific for T cells (CD3, CD4, CD8), B cells (CD19), and macrophages (CD11, F4/80). Fluorescence-activated cell sorting (FACS) was performed using CyAn ADP Analyzer (Beckman Coulter, Brea, CA) and analyzed using FlowJo software (Tree Star, Ashland, OR).

### Urine collection

To ensure sufficient production of urine within the experimental period, in some experiments mice received water mixed with 0.5% methyl cellulose and/or a subcutaneous injection of saline immediately prior to acrylamide exposure. Mice were placed in metabolic cages, and the urine was collected over 90 min. After the collection period, the mice were euthanized, and the urine that was present in the bladder was recovered and added to the respective sample. Urine was centrifuged for 10 min at 16,100 g at 4°C to remove debris and stored at -80°C until ^1^H NMR analysis.

### ^1^H NMR sample preparation and analysis

Prior to ^1^H NMR analysis, 250 μL of urine were added to 5 mm NMR tubes (Wilmad) and mixed with 300 μL sodium phosphate buffer (pH 7.4) in D_2_O with 0.1 mM TSP and 0.05% sodium azide. The ^1^H NMR spectra were acquired on a Bruker Avance II 850 Spectrometer equipped with a TCI H-C/N-D 5 mm CryoProbe. For each sample, 32 scans were collected for a total number of 65,536 points over an acquisition time of 3.2 seconds and a spectral width of 10,204 Hz. The ^1^H NMR spectra were analyzed using ACD/NMR Processor. Raw free induction decay (FID) files were zero filled to 131,072 points, multiplied by 0.3 Hz exponential function, and Fourier transformed. The spectra were referenced to the peak corresponding to TSP at 0 ppm, phased on the mouse phasing setting, and manually baseline corrected. Prior to quantification, the water peaks were removed from the spectra.

## Supporting information

S1 Fig Single family GST knockout mice do not show compensatory hepatic GST expression. Gst transcript levels of (A) female wild-type and ΔM mice and (B) male wild-type and ΔP mice. The tables below each panel show the percent to which each transcript represents its individual family or the total hepatic GST content of wild-type mice (calculated from copy number data in Fig 1A). Data represent means ± SEM; n = 3 (except for Gstt4, where n = 2 for WT in both A and B because overall transcript levels were very low). Data were analyzed by unpaired t-test between genotypes; * p < 0.05; ** p < 0.01.
(PDF)

S2 Fig Loss of GSTT does not affect hepatic GST activity towards CDNB. GST activity towards the substrate CDNB in S9 fractions from male WT and ΔT mice. Data represent means ± S.E.M.; n = 3.
(PDF)

S3 Fig Female ΔPMT mice are more sensitive to acrylamide-induced peripheral neuropathy. Rotarod performance of female mice after acrylamide treatment in drinking water. Treatment was initiated in mice at 2.5–4 weeks of age, with a treatment schedule of 50 ppm for 3 days, 200 ppm for 19–20 days, and a 0 ppm washout period for 3 days prior to behavioral testing. Data represent means ± SEM, n = 9. Data analyzed by t-test; **** p < 0.0001.
(PDF)

S4 Fig Acrylamide does not result in increased plasma ALT levels. Plasma ALT measurements in male mice 24 hours after a single exposure to a single 50 mg/kg i.p. injection of acrylamide. A separate experiment in wild-type mice injected with acetaminophen (6 hours after a single 300 mg/kg i.p. injection) is also included as a positive control for hepatotoxicity. Data represent means ± SEM, n = 3–6. Data analyzed by one-way ANOVA corrected for multiple comparisons (acrylamide) or by unpaired t-test (acetaminophen); *** p < 0.001.
(PDF)

S5 Fig Acrylamide exposure results in decreased liver and spleen sizes, as calculated as percent body weight. Relative liver (A, B) or spleen (C, D) weights of female wild-type and ΔPMT mice 24 hours after two i.p. injections of 50 mg/kg acrylamide once every 24 hours. Organ weights were calculated as percentages relative to initial body weight (A, C, before acrylamide treatment) or relative to final body weight (B, D, after necropsy). Data represent means ± S.E.M.; n = 6. Data analyzed by t-test; * p < 0.05; **#* p < 0.0001.
(PDF)

S6 Fig Acrylamide induces leukopenia in GST-compromised mice. A) Red blood cell counts in female mice exposed to two i.p. injections of 50 mg/kg acrylamide once every 24 hours. Male mice were exposed to this same acrylamide dosing scheme, and spleen weights (B) were measured, in addition to differential cell counts (C), which were obtained through fluorescence-activated cell sorting (FACS) in spleen samples. The FACS sorting shows decreased white blood cells of all types, including macrophages, B cells, and T cells. Data represent means ± SEM, n = 6 (A) and n = 3–4 (B, C). Data were analyzed by one-way ANOVA corrected for multiple comparisons; * p < 0.05; ** p < 0.01; *** p < 0.001.
(PDF)

S7 Fig Acrylamide-induced gastroparesis is both a dose- and strain-dependent effect in wild-type mice. A) Stomach weights do not differ between wild-type and ΔPMT after a single acrylamide injection of 75 mg/kg bw. (B) After a single acrylamide injection of 50 mg/kg bw, 129S6 was the only mouse strain to show gastroparesis. (C) C57BL/6J mice show gastroparesis at doses of 100 mg/kg bw acrylamide. (D) GST-CDNB activity in livers of 129S6 mice is slightly lower than that of C57BL/6J strain. All mice tested were wild-type males (A-C) and females (D). Data represent means ± SEM; n = 3–6; ** p < 0.01, analyzed by unpaired t-test (A) or one-way ANOVA corrected for multiple comparisons (B-D).
(PDF)

S8 Fig Sodium saccharin can be used in ^1^H NMR analysis to normalize urinary acrylamide-derived metabolites. A) Representative ^1^H NMR spectrum of urine collected from an uninjected female mouse. (B) ^1^H NMR spectrum of urine collected 90 minutes after a female mouse was injected with 50 mg/kg saccharin.
(PDF)

S1 Table The Michaelis-Menten parameters show the sex-dependent contribution of individual GST families to CDNB metabolism. Michaelis-Menten parameters for liver GST activity towards CDNB in male and female mice across various genotypes. Statistics represent significant values relative to those in wild-type. Data represent means ± S.E.M.; n = 3; * p < 0.05, ** p < 0.01, **** p < 0.0001, analyzed by one-way ANOVA corrected for multiple comparisons.
(PDF)

S2 Table An analysis of hepatic mRNA transcripts demonstrates no consistent damage response in the liver after acute acrylamide exposure. Fold change (expressed as means ± SEM (n)) of gene expression in the liver relative to PBS controls. Mice were exposed to 2 i.p. injections of 50 mg/kg acrylamide once every 24 hours. *n*.*p*. = experiment not performed for this group. a = * by ANOVA to PBS; b = ** by t test to PBS; c = *** by ANOVA to PBS and WT.
(PDF)

## References

[1] Sheehan D, Meade G, Foley VM, Dowd CA. Structure, function and evolution of glutathione transferases: implications for classification of non-mammalian members of an ancient enzyme superfamily. The Biochemical journal. 2001;360(Pt 1):1–16. Epub 2001/11/07. 10.1042/0264-6021:3600001 11695986 PMC1222196

[2] Hayes JD, Strange RC. Glutathione S-transferase polymorphisms and their biological consequences. Pharmacology. 2000;61(3):154–66. Epub 2000/09/06. 10.1159/000028396 . 10971201

[3] McIlwain CC, Townsend DM, Tew KD. Glutathione S-transferase polymorphisms: cancer incidence and therapy. Oncogene. 2006;25(11):1639–48. Epub 2006/03/22. 10.1038/sj.onc.1209373 16550164 PMC6361140

[4] Chang J, Ma JZ, Zeng Q, Cechova S, Gantz A, Nievergelt C, et al Loss of GSTM1, a NRF2 target, is associated with accelerated progression of hypertensive kidney disease in the African American Study of Kidney Disease (AASK). American journal of physiology Renal physiology. 2013;304(4):F348–55. Epub 2012/12/12. 10.1152/ajprenal.00568.2012 23220723 PMC3566499

[5] Josephy PD. Genetic variations in human glutathione transferase enzymes: significance for pharmacology and toxicology. Human genomics and proteomics: HGP. 2010;2010:876940 Epub 2010/10/29. 10.4061/2010/876940 20981235 PMC2958679

[6] Henderson CJ, Wolf CR. Knockout and transgenic mice in glutathione transferase research. Drug metabolism reviews. 2011;43(2):152–64. Epub 2011/03/24. 10.3109/03602532.2011.562900 . 21425933

[7] Ginsberg G, Smolenski S, Hattis D, Guyton KZ, Johns DO, Sonawane B. Genetic Polymorphism in Glutathione Transferases (GST): Population distribution of GSTM1, T1, and P1 conjugating activity. Journal of toxicology and environmental health Part B, Critical reviews. 2009;12(5–6):389–439. Epub 2010/02/26. 10.1080/10937400903158375 . 20183528

[8] Engle MR, Singh SP, Czernik PJ, Gaddy D, Montague DC, Ceci JD, et al Physiological role of mGSTA4-4, a glutathione S-transferase metabolizing 4-hydroxynonenal: generation and analysis of mGsta4 null mouse. Toxicology and applied pharmacology. 2004;194(3):296–308. Epub 2004/02/06. 10.1016/j.taap.2003.10.001 . 14761685

[9] Henderson CJ, Wolf CR, Kitteringham N, Powell H, Otto D, Park BK. Increased resistance to acetaminophen hepatotoxicity in mice lacking glutathione S-transferase Pi. Proceedings of the National Academy of Sciences of the United States of America. 2000;97(23):12741–5. Epub 2000/11/01. 10.1073/pnas.220176997 11058152 PMC18834

[10] McMillan DH, van der Velden JL, Lahue KG, Qian X, Schneider RW, Iberg MS, et al Attenuation of lung fibrosis in mice with a clinically relevant inhibitor of glutathione-S-transferase pi. JCI insight. 2016;1(8). Epub 2016/07/01. 10.1172/jci.insight.85717 27358914 PMC4922427

[11] Oakley AJ, Lo Bello M, Nuccetelli M, Mazzetti AP, Parker MW. The ligandin (non-substrate) binding site of human Pi class glutathione transferase is located in the electrophile binding site (H-site). Journal of molecular biology. 1999;291(4):913–26. Epub 1999/08/24. 10.1006/jmbi.1999.3029 . 10452896

[12] Townsend DM, Manevich Y, He L, Hutchens S, Pazoles CJ, Tew KD. Novel role for glutathione S-transferase pi. Regulator of protein S-Glutathionylation following oxidative and nitrosative stress. The Journal of biological chemistry. 2009;284(1):436–45. Epub 2008/11/08. 10.1074/jbc.M805586200 18990698 PMC2610519

[13] Zhang J, Grek C, Ye ZW, Manevich Y, Tew KD, Townsend DM. Pleiotropic functions of glutathione S-transferase P. Advances in cancer research. 2014;122:143–75. Epub 2014/06/30. 10.1016/B978-0-12-420117-0.00004-9 24974181 PMC5079281

[14] Tew KD, Manevich Y, Grek C, Xiong Y, Uys J, Townsend DM. The role of glutathione S-transferase P in signaling pathways and S-glutathionylation in cancer. Free radical biology & medicine. 2011;51(2):299–313. Epub 2011/05/12. 10.1016/j.freeradbiomed.2011.04.013 21558000 PMC3125017

[15] Jones JT, Qian X, van der Velden JL, Chia SB, McMillan DH, Flemer S, et al Glutathione S-transferase pi modulates NF-kappaB activation and pro-inflammatory responses in lung epithelial cells. Redox biology. 2016;8:375–82. Epub 2016/04/09. 10.1016/j.redox.2016.03.005 27058114 PMC4827796

[16] Xiang Z, Snouwaert JN, Kovarova M, Nguyen M, Repenning PW, Latour AM, et al Mice lacking three Loci encoding 14 glutathione transferase genes: a novel tool for assigning function to the GSTP, GSTM, and GSTT families. Drug metabolism and disposition: the biological fate of chemicals. 2014;42(6):1074–83. Epub 2014/03/25. 10.1124/dmd.113.056481 24658454 PMC4014662

[17] Calleman CJ, Wu Y, He F, Tian G, Bergmark E, Zhang S, et al Relationships between biomarkers of exposure and neurological effects in a group of workers exposed to acrylamide. Toxicology and applied pharmacology. 1994;126(2):361–71. Epub 1994/06/01. 10.1006/taap.1994.1127 . 8209389

[18] Tareke E, Rydberg P, Karlsson P, Eriksson S, Tornqvist M. Analysis of acrylamide, a carcinogen formed in heated foodstuffs. Journal of agricultural and food chemistry. 2002;50(17):4998–5006. Epub 2002/08/09. 10.1021/jf020302f . 12166997

[19] Organization WH. Health implications of acrylamide in food. 2002.

[20] Lineback DR, Coughlin JR, Stadler RH. Acrylamide in foods: a review of the science and future considerations. Annual review of food science and technology. 2012;3:15–35. Epub 2011/12/06. 10.1146/annurev-food-022811-101114 . 22136129

[21] Virk-Baker MK, Nagy TR, Barnes S, Groopman J. Dietary acrylamide and human cancer: a systematic review of literature. Nutrition and cancer. 2014;66(5):774–90. Epub 2014/05/31. 10.1080/01635581.2014.916323 PubMed Central PMCID: PMC4164905. 24875401 PMC4164905

[22] Twaddle NC, Churchwell MI, McDaniel LP, Doerge DR. Autoclave sterilization produces acrylamide in rodent diets: implications for toxicity testing. Journal of agricultural and food chemistry. 2004;52(13):4344–9. Epub 2004/06/24. 10.1021/jf0497657 . 15212490

[23] Sumner SCJ, MacNeela JP, Fennell TR. Characterization and quantitation of urinary metabolites of [1,2,3-13C]acrylamide in rats and mice using carbon-13 nuclear magnetic resonance spectroscopy. Chemical Research in Toxicology. 1992;5(1):81–9. 10.1021/tx00025a014 1581543

[24] Fennell TR, Sumner SC, Snyder RW, Burgess J, Friedman MA. Kinetics of elimination of urinary metabolites of acrylamide in humans. Toxicological sciences: an official journal of the Society of Toxicology. 2006;93(2):256–67. Epub 2006/07/28. 10.1093/toxsci/kfl069 . 16870689

[25] Fennell TR, Sumner SC, Snyder RW, Burgess J, Spicer R, Bridson WE, et al Metabolism and hemoglobin adduct formation of acrylamide in humans. Toxicological sciences: an official journal of the Society of Toxicology. 2005;85(1):447–59. Epub 2004/12/31. 10.1093/toxsci/kfi069 . 15625188

[26] Knight TR, Choudhuri S, Klaassen CD. Constitutive mRNA expression of various glutathione S-transferase isoforms in different tissues of mice. Toxicological sciences: an official journal of the Society of Toxicology. 2007;100(2):513–24. Epub 2007/09/25. 10.1093/toxsci/kfm233 . 17890767

[27] Hayes JD, Flanagan JU, Jowsey IR. Glutathione transferases. Annual review of pharmacology and toxicology. 2005;45:51–88. Epub 2005/04/12. 10.1146/annurev.pharmtox.45.120403.095857 . 15822171

[28] Baars AJ, Jansen M, Breimer DD. The influence of phenobarbital, 3-methylcholanthrene and 2,3,7,8-tetrachlorodibenzo-p-dioxin on glutathione S-transferase activity of rat liver cytosol. Biochemical pharmacology. 1978;27(21):2487–97. Epub 1978/01/01. 10.1016/0006-2952(78)90314-3 . 728202

[29] Jemth P, Mannervik B. Kinetic characterization of recombinant human glutathione transferase T1-1, a polymorphic detoxication enzyme. Archives of biochemistry and biophysics. 1997;348(2):247–54. Epub 1998/01/22. 10.1006/abbi.1997.0357 . 9434735

[30] Ghanayem BI, Witt KL, Kissling GE, Tice RR, Recio L. Absence of acrylamide-induced genotoxicity in CYP2E1-null mice: evidence consistent with a glycidamide-mediated effect. Mutation research. 2005;578(1–2):284–97. Epub 2005/06/29. 10.1016/j.mrfmmm.2005.05.004 . 15982677

[31] Gamboa da Costa G, Churchwell MI, Hamilton LP, Von Tungeln LS, Beland FA, Marques MM, et al DNA adduct formation from acrylamide via conversion to glycidamide in adult and neonatal mice. Chem Res Toxicol. 2003;16(10):1328–37. Epub 2003/10/21. 10.1021/tx034108e . 14565774

[32] Barber DS, Hunt JR, Ehrich MF, Lehning EJ, LoPachin RM. Metabolism, toxicokinetics and hemoglobin adduct formation in rats following subacute and subchronic acrylamide dosing. Neurotoxicology. 2001;22(3):341–53. Epub 2001/07/18. 10.1016/s0161-813x(01)00024-9 . 11456335

[33] Brat DJ, Brimijoin S. Acrylamide and glycidamide impair neurite outgrowth in differentiating N1E.115 neuroblastoma without disturbing rapid bidirectional transport of organelles observed by video microscopy. Journal of neurochemistry. 1993;60(6):2145–52. Epub 1993/06/01. 10.1111/j.1471-4159.1993.tb03499.x . 8492122

[34] Costa LG, Deng H, Calleman CJ, Bergmark E. Evaluation of the neurotoxicity of glycidamide, an epoxide metabolite of acrylamide: behavioral, neurochemical and morphological studies. Toxicology. 1995;98(1–3):151–61. Epub 1995/04/12. 10.1016/0300-483x(94)02986-5 . 7740544

[35] Gilbert SG, Maurissen JP. Assessment of the effects of acrylamide, methylmercury, and 2,5-hexanedione on motor functions in mice. Journal of toxicology and environmental health. 1982;10(1):31–41. Epub 1982/07/01. 10.1080/15287398209530228 . 7131587

[36] Ko MH, Chen WP, Lin-Shiau SY, Hsieh ST. Age-dependent acrylamide neurotoxicity in mice: morphology, physiology, and function. Experimental neurology. 1999;158(1):37–46. Epub 1999/08/17. 10.1006/exnr.1999.7102 . 10448416

[37] Von Burg R, Penney DP, Conroy PJ. Acrylamide neurotoxicity in the mouse: a behavioral, electrophysiological and morphological study. Journal of applied toxicology: JAT. 1981;1(4):227–33. Epub 1981/08/01. 10.1002/jat.2550010409 . 7184942

[38] Shelby MD, Cain KT, Hughes LA, Braden PW, Generoso WM. Dominant lethal effects of acrylamide in male mice. Mutation research. 1986;173(1):35–40. Epub 1986/01/01. 10.1016/0165-7992(86)90008-4 . 3941677

[39] Yoon E, Babar A, Choudhary M, Kutner M, Pyrsopoulos N. Acetaminophen-Induced Hepatotoxicity: a Comprehensive Update. Journal of clinical and translational hepatology. 2016;4(2):131–42. Epub 2016/06/29. 10.14218/JCTH.2015.00052 27350943 PMC4913076

[40] Zaidi SI, Raisuddin S, Singh KP, Jafri A, Husain R, Husain MM, et al Acrylamide induced immunosuppression in rats and its modulation by 6-MFA, an interferon inducer. Immunopharmacology and immunotoxicology. 1994;16(2):247–60. Epub 1994/05/01. 10.3109/08923979409007093 . 8077609

[41] Doerge DR, Young JF, McDaniel LP, Twaddle NC, Churchwell MI. Toxicokinetics of acrylamide and glycidamide in B6C3F1 mice. Toxicology and applied pharmacology. 2005;202(3):258–67. Epub 2005/01/26. 10.1016/j.taap.2004.07.001 . 15667831

[42] Sun J, Schnackenberg LK, Pence L, Bhattacharyya S, Doerge DR, Bowyer JF, et al Metabolomic analysis of urine from rats chronically dosed with acrylamide using NMR and LC/MS. Metabolomics. 2010;6(4):550–63. 10.1007/s11306-010-0225-8

[43] Lethco EJ, Wallace WC. The metabolism of saccharin in animals. Toxicology. 1975;3(3):287–300. Epub 1975/01/01. 10.1016/0300-483x(75)90030-x . 1092032

[44] Byard JL, Goldberg L. The metabolism of saccharin in laboratory animals. Food and cosmetics toxicology. 1973;11(3):391–402. Epub 1973/06/01. 10.1016/0015-6264(73)90005-9 . 4199497

[45] Testa B, Kramer SD. The biochemistry of drug metabolism—an introduction: part 4. reactions of conjugation and their enzymes. Chemistry & biodiversity. 2008;5(11):2171–336. Epub 2008/11/28. 10.1002/cbdv.200890199 . 19035562

[46] Doerge DR, Twaddle NC, Boettcher MI, McDaniel LP, Angerer J. Urinary excretion of acrylamide and metabolites in Fischer 344 rats and B6C3F(1) mice administered a single dose of acrylamide. Toxicology letters. 2007;169(1):34–42. Epub 2007/01/17. 10.1016/j.toxlet.2006.12.002 . 17224249

[47] Dixit R, Mukhtar H, Seth PK, Murti CR. Binding of acrylamide with glutathione-S-transferases. Chemico-biological interactions. 1980;32(3):353–9. Epub 1980/11/01. 10.1016/0009-2797(80)90103-9 . 7428122

[48] Dixit R, Mukhtar H, Seth PK, Murti CR. Conjugation of acrylamide with glutathione catalysed by glutathione-S-transferases of rat liver and brain. Biochemical pharmacology. 1981;30(13):1739–44. Epub 1981/07/01. 10.1016/0006-2952(81)90003-4 . 7271861

[49] Sumner SC, Williams CC, Snyder RW, Krol WL, Asgharian B, Fennell TR. Acrylamide: a comparison of metabolism and hemoglobin adducts in rodents following dermal, intraperitoneal, oral, or inhalation exposure. Toxicological sciences: an official journal of the Society of Toxicology. 2003;75(2):260–70. Epub 2003/07/29. 10.1093/toxsci/kfg191 . 12883088

[50] Abel EL, Opp SM, Verlinde CL, Bammler TK, Eaton DL. Characterization of atrazine biotransformation by human and murine glutathione S-transferases. Toxicological sciences: an official journal of the Society of Toxicology. 2004;80(2):230–8. Epub 2004/04/30. 10.1093/toxsci/kfh152 . 15115887

[51] Seidegard J, Vorachek WR, Pero RW, Pearson WR. Hereditary differences in the expression of the human glutathione transferase active on trans-stilbene oxide are due to a gene deletion. Proceedings of the National Academy of Sciences of the United States of America. 1988;85(19):7293–7. Epub 1988/10/01. 10.1073/pnas.85.19.7293 3174634 PMC282172

[52] Adler V, Yin Z, Fuchs SY, Benezra M, Rosario L, Tew KD, et al Regulation of JNK signaling by GSTp. The EMBO journal. 1999;18(5):1321–34. Epub 1999/03/04. 10.1093/emboj/18.5.1321 10064598 PMC1171222

[53] Zhang J, Ye ZW, Singh S, Townsend DM, Tew KD. An evolving understanding of the S-glutathionylation cycle in pathways of redox regulation. Free radical biology & medicine. 2018;120:204–16. Epub 2018/03/27. 10.1016/j.freeradbiomed.2018.03.038 29578070 PMC5940525

[54] Martyniuk CJ, Feswick A, Fang B, Koomen JM, Barber DS, Gavin T, et al Protein targets of acrylamide adduct formation in cultured rat dopaminergic cells. Toxicology letters. 2013;219(3):279–87. Epub 2013/04/10. 10.1016/j.toxlet.2013.03.031 23566896 PMC3707521

[55] University of North Carolina at Chapel Hill Standard on Humane Endpoints in Rodents: UNC IACUC; 2017. Available from: https://research.unc.edu/files/2012/11/Guidelines-for-Humane-Endpoints-of-Rodents.pdf.

[56] Habig WH, Pabst MJ, Jakoby WB. Glutathione S-transferases. The first enzymatic step in mercapturic acid formation. The Journal of biological chemistry. 1974;249(22):7130–9. Epub 1974/11/25. . 4436300

[57] Tice RR, Agurell E, Anderson D, Burlinson B, Hartmann A, Kobayashi H, et al Single cell gel/comet assay: guidelines for in vitro and in vivo genetic toxicology testing. Environmental and molecular mutagenesis. 2000;35(3):206–21. Epub 2000/03/29. 10.1002/(sici)1098-2280(2000)35:3<206::aid-em8>3.0.co;2-j . 10737956

[58] Gyori BM, Venkatachalam G, Thiagarajan PS, Hsu D, Clement MV. OpenComet: an automated tool for comet assay image analysis. Redox biology. 2014;2:457–65. Epub 2014/03/14. 10.1016/j.redox.2013.12.020 24624335 PMC3949099

