# Supplementary figures and images for: Beyond detoxification: Pleiotropic functions of multiple glutathione S-transferase isoforms protect mice against a toxic electrophile
Source: PLoS One. 2019 Nov 20;14(11):e0225449. doi: 10.1371/journal.pone.0225449 (PMC6867637; doi:10.1371/journal.pone.0225449)

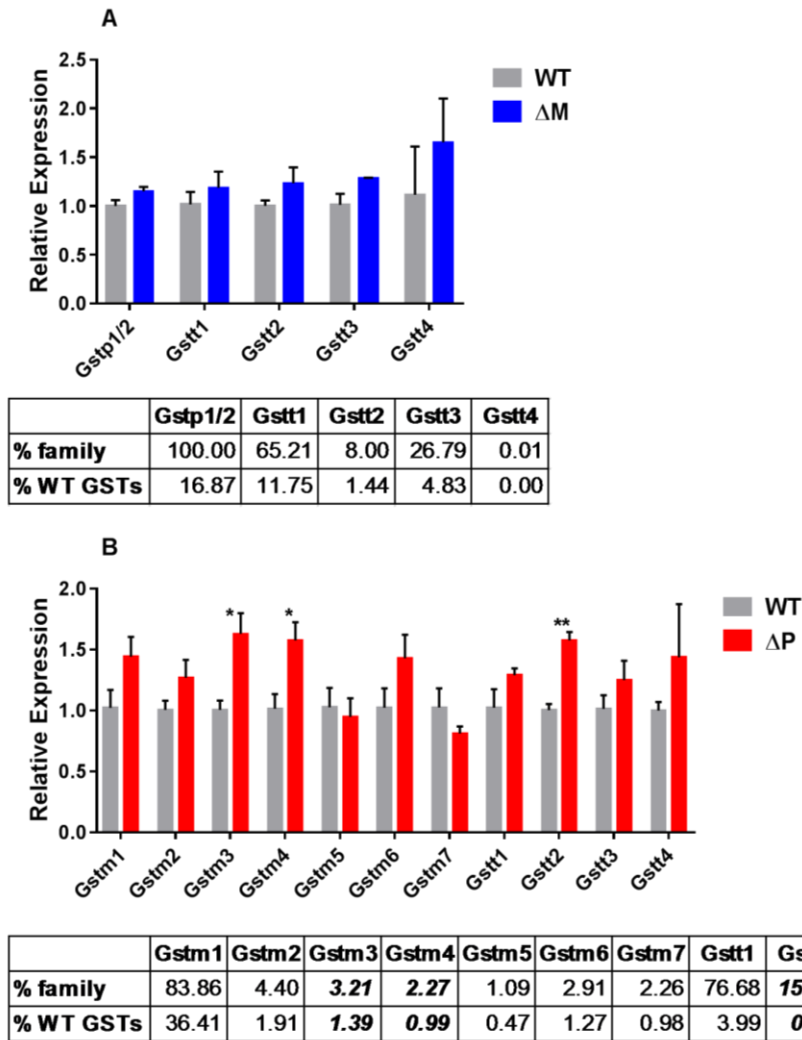

**S1 Figure. Single family GST knockout mice do not show compensatory hepatic GST expression.**

Supplement: S1 Fig — Gst transcript levels of (A) female wild-type and ΔM mice and (B) male wild-type and ΔP mice. The tables below each panel show the percent to which each transcript represents its individual family or the total hepatic GST content of wild-type mice (calculated from copy number data in Fig 1A). Data represent means ± SEM; n = 3 (except for Gstt4, where n = 2 for WT in both A and B because overall transcript levels were very low). Data were analyzed by unpaired t-test between genotypes; * p < 0.05; ** p < 0.01. (PDF) [file pone.0225449.s001.pdf]

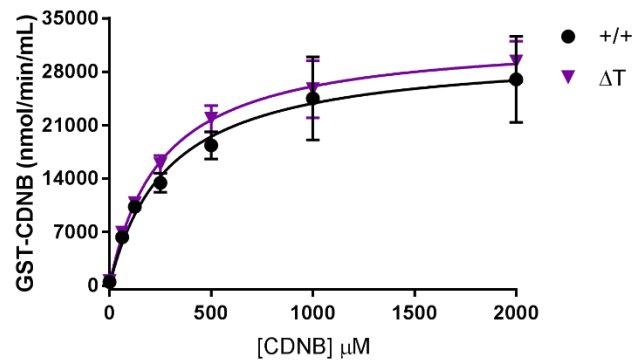

**S2 Figure. Loss of GSTT does not affect hepatic GST activity towards CDNB.**

Supplement: S2 Fig — GST activity towards the substrate CDNB in S9 fractions from male WT and ΔT mice. Data represent means ± S.E.M.; n = 3. (PDF) [file pone.0225449.s002.pdf]

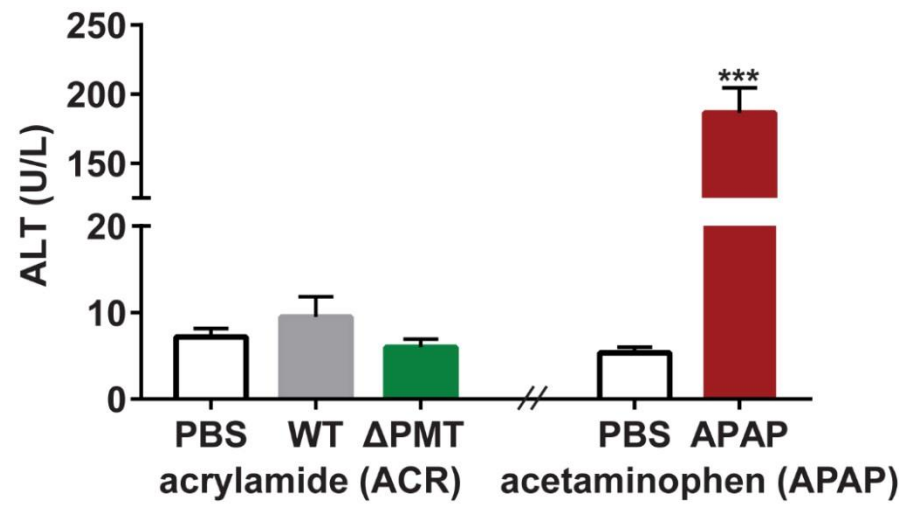

S4 Figure. Acrylamide does not result in increased plasma ALT levels.

Supplement: S4 Fig — Plasma ALT measurements in male mice 24 hours after a single exposure to a single 50 mg/kg i.p. injection of acrylamide. A separate experiment in wild-type mice injected with acetaminophen (6 hours after a single 300 mg/kg i.p. injection) is also included as a positive control for hepatotoxicity. Data represent means ± SEM, n = 3–6. Data analyzed by one-way ANOVA corrected for multiple comparisons (acrylamide) or by unpaired t-test (acetaminophen); *** p < 0.001. (PDF) [file pone.0225449.s004.pdf]

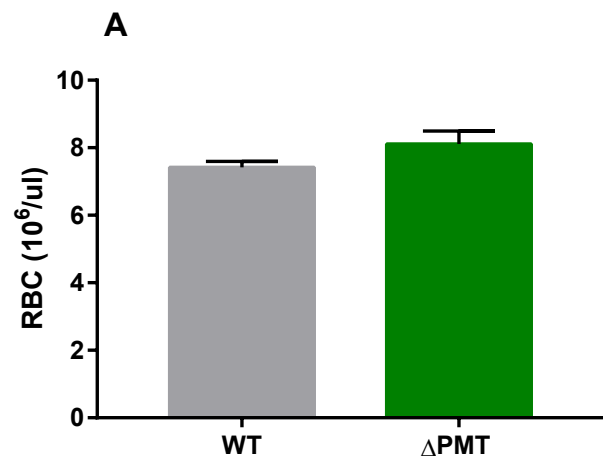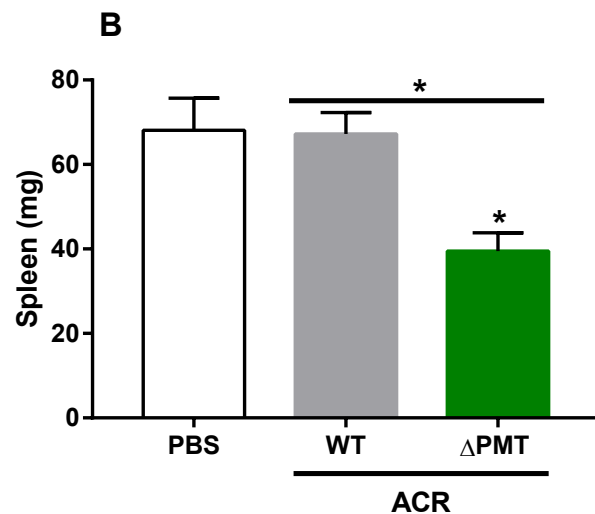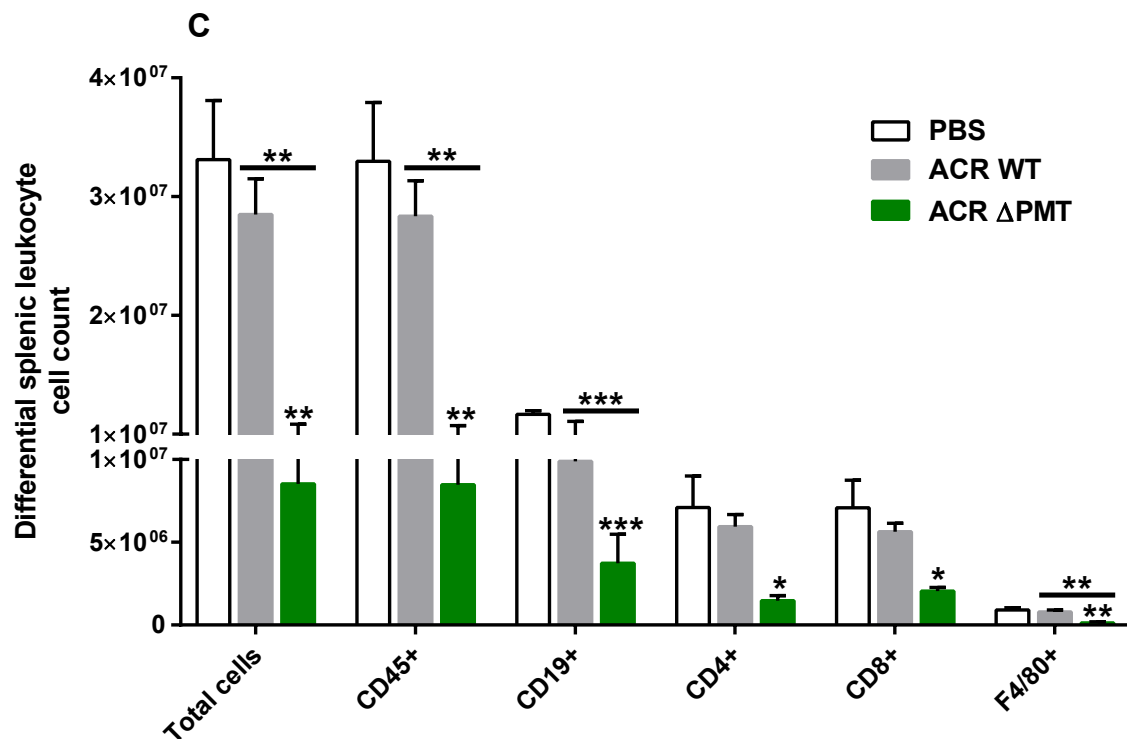

Supplement: S6 Fig — A) Red blood cell counts in female mice exposed to two i.p. injections of 50 mg/kg acrylamide once every 24 hours. Male mice were exposed to this same acrylamide dosing scheme, and spleen weights (B) were measured, in addition to differential cell counts (C), which were obtained through fluorescence-activated cell sorting (FACS) in spleen samples. The FACS sorting shows decreased white blood cells of all types, including macrophages, B cells, and T cells. Data represent means ± SEM, n = 6 (A) and n = 3–4 (B, C). Data were analyzed by one-way ANOVA corrected for multiple comparisons; * p < 0.05; ** p < 0.01; *** p < 0.001. (PDF) [file pone.0225449.s006.pdf]
