# Supplementary material for: Beyond detoxification: Pleiotropic functions of multiple glutathione S-transferase isoforms protect mice against a toxic electrophile
Source: PLoS One. 2019 Nov 20;14(11):e0225449. doi: 10.1371/journal.pone.0225449 (PMC6867637; doi:10.1371/journal.pone.0225449)
Supplement: S3 Fig — Rotarod performance of female mice after acrylamide treatment in drinking water. Treatment was initiated in mice at 2.5–4 weeks of age, with a treatment schedule of 50 ppm for 3 days, 200 ppm for 19–20 days, and a 0 ppm washout period for 3 days prior to behavioral testing. Data represent means ± SEM, n = 9. Data analyzed by t-test; **** p < 0.0001. (PDF) [file pone.0225449.s003.pdf]

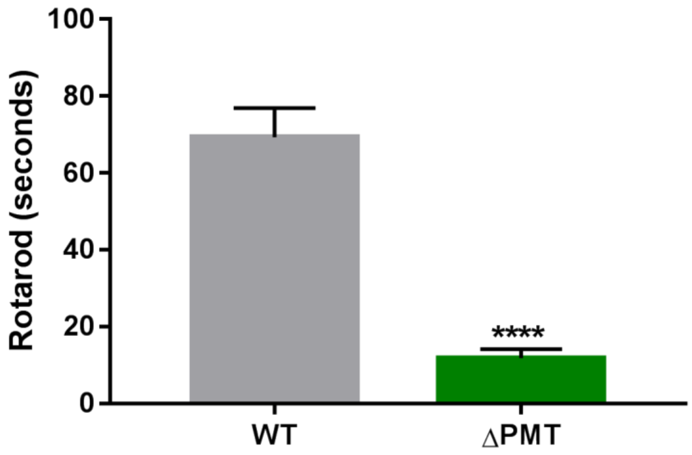

S3 Figure. Female ΔPMT mice are more sensitive to acrylamide-induced peripheral neuropathy.
