# Supplementary material for: Beyond detoxification: Pleiotropic functions of multiple glutathione S-transferase isoforms protect mice against a toxic electrophile
Source: PLoS One. 2019 Nov 20;14(11):e0225449. doi: 10.1371/journal.pone.0225449 (PMC6867637; doi:10.1371/journal.pone.0225449)
Supplement: S5 Fig — Relative liver (A, B) or spleen (C, D) weights of female wild-type and ΔPMT mice 24 hours after two i.p. injections of 50 mg/kg acrylamide once every 24 hours. Organ weights were calculated as percentages relative to initial body weight (A, C, before acrylamide treatment) or relative to final body weight (B, D, after necropsy). Data represent means ± S.E.M.; n = 6. Data analyzed by t-test; * p < 0.05; **#* p < 0.0001. (PDF) [file pone.0225449.s005.pdf]

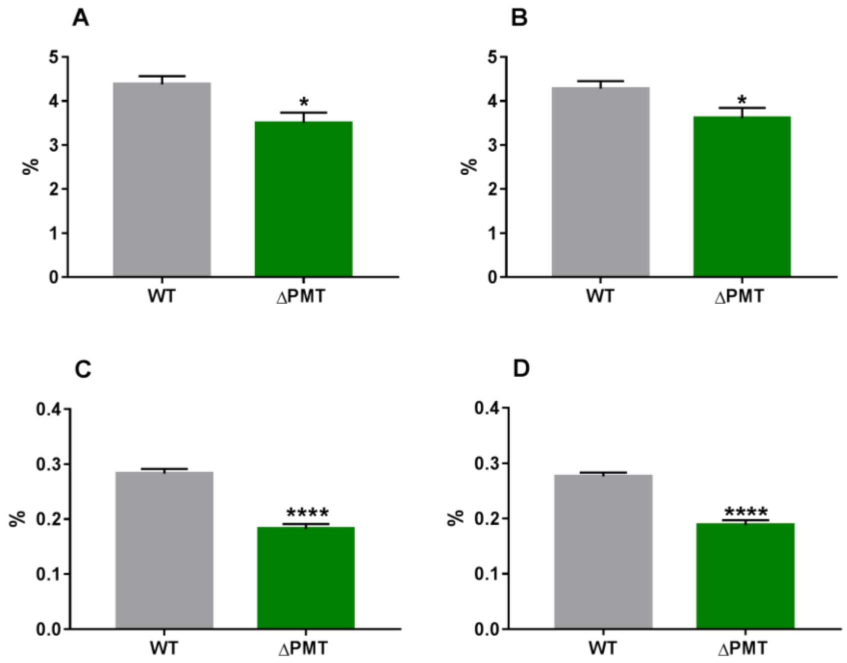

S5 Figure. Acrylamide exposure results in decreased liver and spleen sizes, as calculated as percent body weight.
