# Supplementary material for: Beyond detoxification: Pleiotropic functions of multiple glutathione S-transferase isoforms protect mice against a toxic electrophile
Source: PLoS One. 2019 Nov 20;14(11):e0225449. doi: 10.1371/journal.pone.0225449 (PMC6867637; doi:10.1371/journal.pone.0225449)
Supplement: S7 Fig — A) Stomach weights do not differ between wild-type and ΔPMT after a single acrylamide injection of 75 mg/kg bw. (B) After a single acrylamide injection of 50 mg/kg bw, 129S6 was the only mouse strain to show gastroparesis. (C) C57BL/6J mice show gastroparesis at doses of 100 mg/kg bw acrylamide. (D) GST-CDNB activity in livers of 129S6 mice is slightly lower than that of C57BL/6J strain. All mice tested were wild-type males (A-C) and females (D). Data represent means ± SEM; n = 3–6; ** p < 0.01, analyzed by unpaired t-test (A) or one-way ANOVA corrected for multiple comparisons (B-D). (PDF) [file pone.0225449.s007.pdf]

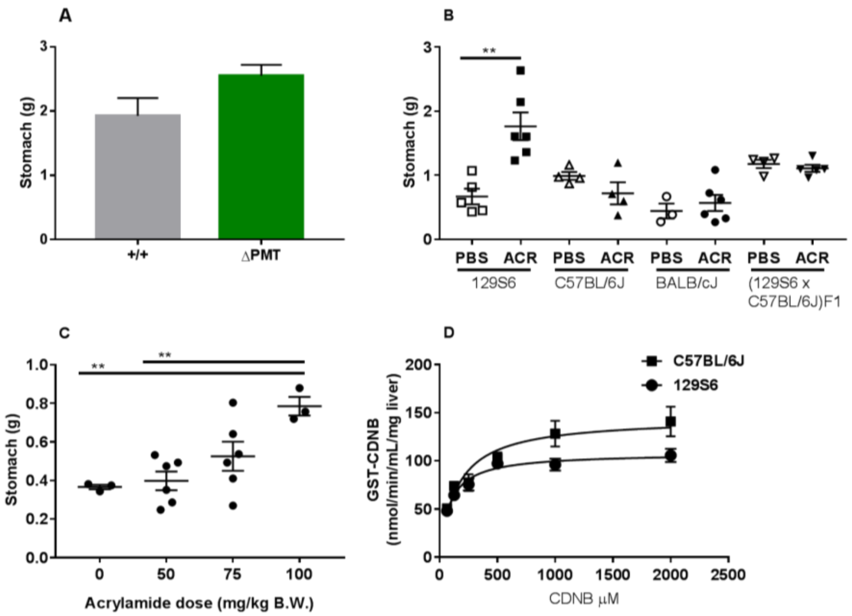

S7 Figure. Acrylamide-induced gastroparesis is both a dose- and strain-dependent effect in wild-type mice.
