# Supplementary material for: Beyond detoxification: Pleiotropic functions of multiple glutathione S-transferase isoforms protect mice against a toxic electrophile
Source: PLoS One. 2019 Nov 20;14(11):e0225449. doi: 10.1371/journal.pone.0225449 (PMC6867637; doi:10.1371/journal.pone.0225449)
Supplement: S8 Fig — A) Representative 1H NMR spectrum of urine collected from an uninjected female mouse. (B) 1H NMR spectrum of urine collected 90 minutes after a female mouse was injected with 50 mg/kg saccharin. (PDF) [file pone.0225449.s008.pdf]

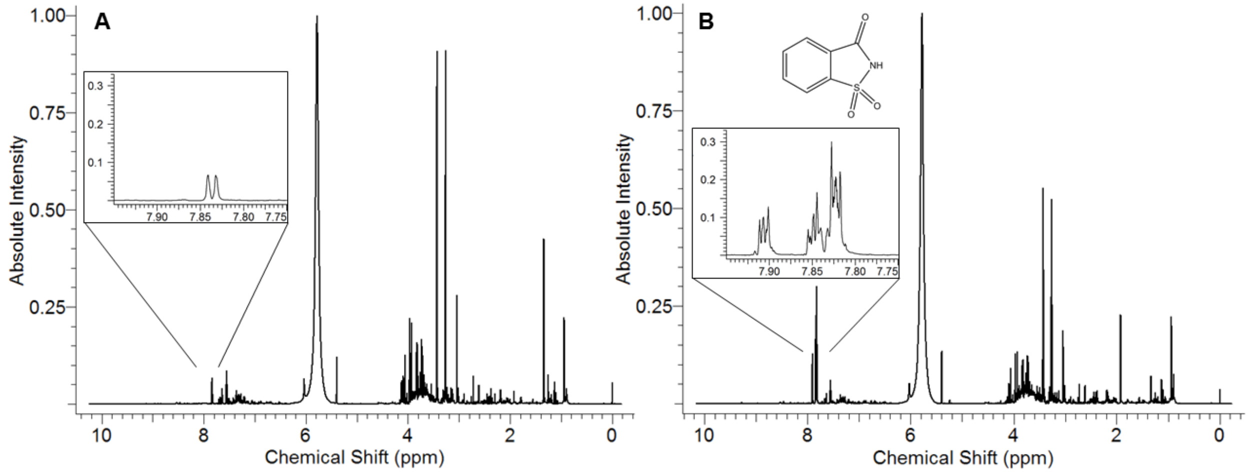

Figure S8. Sodium saccharin can be used in ^{1}H NMR analysis to normalize urinary acrylamide-derived metabolites.
