# Supplementary material for: Beyond detoxification: Pleiotropic functions of multiple glutathione S-transferase isoforms protect mice against a toxic electrophile
Source: PLoS One. 2019 Nov 20;14(11):e0225449. doi: 10.1371/journal.pone.0225449 (PMC6867637; doi:10.1371/journal.pone.0225449)
Supplement: S1 Table — Michaelis-Menten parameters for liver GST activity towards CDNB in male and female mice across various genotypes. Statistics represent significant values relative to those in wild-type. Data represent means ± S.E.M.; n = 3; * p < 0.05, ** p < 0.01, **** p < 0.0001, analyzed by one-way ANOVA corrected for multiple comparisons. (PDF) [file pone.0225449.s009.pdf]

	Females					Males				
Geno	V_{max} (nmol/min/mL/mg liver)	p	K_m (µM CDNB)	p	R^2	V_{max} (nmol/min/mL/mg liver)	p	K_m (µM CDNB)	p	R^2
+/+	141.5 ± 6.539		102.7 ± 20.04		0.84	207.5 ± 14.03		288.1 ± 59.79		0.92
ΔM	**48.4 ± 3.09**	****	**427.3 ± 74.36**	*	0.94	212.2 ± 13.16	n.s.	593.3 ± 90.51	n.s.	0.97
ΔP	**119.9 ± 4.661**	*	80.45 ± 14.43	n.s.	0.85	**131.6 ± 2.91**	**	105.1 ± 9.75	n.s.	0.98
ΔPMT	**24.54 ± 2.118**	****	269.8 ± 72.84	n.s.	0.84	**23.61 ± 1.84**	****	**678.2 ± 124.4**	*	0.95

S1 Table. The Michaelis-Menten parameters show the sex-dependent contribution of individual GST families to CDNB metabolism.
