# Supplementary material for: Beyond detoxification: Pleiotropic functions of multiple glutathione S-transferase isoforms protect mice against a toxic electrophile
Source: PLoS One. 2019 Nov 20;14(11):e0225449. doi: 10.1371/journal.pone.0225449 (PMC6867637; doi:10.1371/journal.pone.0225449)
Supplement: S2 Table — Fold change (expressed as means ± SEM (n)) of gene expression in the liver relative to PBS controls. Mice were exposed to 2 i.p. injections of 50 mg/kg acrylamide once every 24 hours. n.p. = experiment not performed for this group. a = * by ANOVA to PBS; b = ** by t test to PBS; c = *** by ANOVA to PBS and WT. (PDF) [file pone.0225449.s010.pdf]

	Females		Males	
Gene	+/+	ΔPMT	+/+	ΔPMT
Il-1β	2.1 ± 0.31 (6)	**2.9 ± 0.71 (6)** [a]	2.2 ± 1.70 (6)	0.3 ± 0.06 (6)
Il1ra	0.9 ± 0.16 (6)	3.8 ± 1.63 (5)	1.4 ± 0.35 (6)	1.5 ± 0.79 (6)
Il-6	0.8 ± 0.13 (5)	8.6 ± 4.68 (4)	2.3 ± 1.97 (6)	1.9 ± 0.61 (6)
Tnf-α	*n.p.*	1.9 ± 0.83 (4)	5.0 ± 4.11 (6)	0.1 ± 0.02 (6)
Hmox1	*n.p.*	1.5 ± 0.53 (4)	0.9 ± 0.20 (4)	**7.4 ± 1.20 (4)** [c]
Nqo1	*n.p.*	1.7 ± 0.34 (4)	1.0 ± 0.25 (4)	1.7 ± 0.23 (4)
Apcs	5.1 ± 1.44 (4)	6.9 ± 2.09 (4)	2.3 ± 0.71 (4)	1.3 ± 0.73 (4)
Gpx	*n.p.*	**0.33 ± 0.09 (4)** [b]	*n.p.*	*n.p.*
Lpin	*n.p.*	0.5 ± 0.08 (4)	*n.p.*	*n.p.*

S2 Table. An analysis of hepatic mRNA transcripts demonstrates no consistent damage response in the liver after acute acrylamide exposure.
